# Persistent effects of intramammary ceftiofur treatment on the gut microbiome and antibiotic resistance in dairy cattle

**DOI:** 10.1186/s42523-023-00274-4

**Published:** 2023-11-09

**Authors:** Karla A. Vasco, Samantha Carbonell, Rebekah E. Sloup, Bailey Bowcutt, Rita R. Colwell, Karlis Graubics, Ronald Erskine, Bo Norby, Pamela L. Ruegg, Lixin Zhang, Shannon D. Manning

**Affiliations:** 1 Department of Microbiology and Molecular Genetics, Michigan State University, E. Lansing, MI 48824 USA; 2 University of Maryland, Institute for Advanced Computer Studies, College Park, MD 20742 USA; 3 Cosmos ID, Inc, Germantown, MD 20874 USA; 4 Department of Large Animal Clinical Sciences, Michigan State University, E. Lansing, MI 48824 USA; 5 Department of Epidemiology and Biostatistics, Michigan State University, E. Lansing, MI 48824 USA

**Keywords:** Cattle, Mastitis, Antibiotic resistance, Microbiome, Resistome, Ceftiofur, beta-lactam

## Abstract

**Background:**

Intramammary (IMM) ceftiofur treatment is commonly used in dairy farms to prevent mastitis, though its impact on the cattle gut microbiome and selection of antibiotic-resistant bacteria has not been elucidated. Herein, we enrolled 40 dairy (Holstein) cows at the end of the lactation phase for dry-cow therapy: 20 were treated with IMM ceftiofur (Spectramast®DC) and a non-antibiotic internal teat sealant (bismuth subnitrate) and 20 (controls) received only bismuth subnitrate. Fecal grab samples were collected before and after treatment (weeks 1, 2, 3, 5, 7, and 9) for bacterial quantification and metagenomic next-generation sequencing.

**Results:**

Overall, 90% and 24% of the 278 samples had Gram-negative bacteria with resistance to ampicillin and ceftiofur, respectively. Most of the cows treated with ceftiofur did not have an increase in the number of resistant bacteria; however, a subset (25%) shed higher levels of ceftiofur-resistant bacteria for up to 2 weeks post-treatment. At week 5, the antibiotic-treated cows had lower microbiota abundance and richness, whereas a greater abundance of genes encoding extended-spectrum β-lactamases (ESBLs), CfxA, ACI-1, and CMY, was observed at weeks 1, 5 and 9. Moreover, the contig and network analyses detected associations between β-lactam resistance genes and phages, mobile genetic elements, and specific genera. Commensal bacterial populations belonging to Bacteroidetes most commonly possessed ESBL genes followed by members of Enterobacteriaceae.

**Conclusion:**

This study highlights variable, persistent effects of IMM ceftiofur treatment on the gut microbiome and resistome in dairy cattle. Antibiotic-treated cattle had an increased abundance of specific taxa and genes encoding ESBL production that persisted for 9 weeks. Fecal shedding of ESBL-producing Enterobacteriaceae, which was classified as a serious public health threat, varied across animals. Together, these findings highlight the need for additional studies aimed at identifying factors associated with shedding levels and the dissemination and persistence of antibiotic resistance determinants on dairy farms across geographic locations.

**Supplementary Information:**

The online version contains supplementary material available at 10.1186/s42523-023-00274-4.

## Background

Globally, multi-drug resistant (MDR) bacteria were estimated to cause 4.95 (3.62–6.57) million deaths a year, with third-generation cephalosporin-resistant *Escherichia coli* and *Klebsiella pneumoniae* among the leading causes of MDR deaths worldwide [1]. These resistant bacterial populations are also considered to be the most concerning and economically impactful antimicrobial-resistant threats in the U.S. [2]. Enterobacteriaceae with resistance to third-generation cephalosporins carry genes encoding extended-spectrum β-lactamase (ESBL) production, which also confer resistance to penicillins and monobactams. Hence, use of third-generation cephalosporins to treat humans and for livestock production may contribute to the emergence of ESBL-producing Enterobacteriaceae. In the U.S, less than 1% of all antibiotics used in livestock correspond to cephalosporins, with most use (80%) occurring in cattle [3]. At present, two cephalosporins are approved for use in dairy cattle and include cephapirin (a first generation cephalosporin) and ceftiofur (a third-generation cephalosporin) [3, 4]. Ceftiofur is approved for use only via the parenteral and intramammary route for therapeutic indications including mastitis, metritis, respiratory disease, and foot rot [4].

Mastitis, an infection of the mammary gland, is the disease with the highest incidence in dairy cattle [5]; hence, ~ 90% of dairy farms use intramammary (IMM) β-lactam antibiotics during the dry-off period to treat and prevent mastitis [5–7]. More specifically, a study of 37 Wisconsin dairy farms reported ceftiofur to be the most common β-lactam antibiotic used intramammarily to treat clinical mastitis and for prophylactic dry-cow therapy [5]. Ceftiofur has bactericidal activity against both Gram-negative and Gram-positive bacterial populations, low toxicity potential, and efficient penetration of most body fluids. Consequently, β-lactams are also used to treat a variety of pathologies in humans such as septicemia, urinary tract infections, respiratory infections, meningitis, and peritonitis.

Cephalosporins like ceftiofur exhibit varied pharmacokinetics and pharmacodynamics based on the route of administration in dairy cattle. When administered parenterally, these drugs rapidly disseminate throughout the body, primarily getting eliminated through the kidneys (61–77%) within 24 h post-administration [8]. Active metabolites of ceftiofur have been detected in the biliary system (~ 30%), [8] ileum, and colon (20% of plasmatic concentration) [9]. Following parenteral application, the half-life of ceftiofur is usually a few hours, though it may vary depending on the animal’s health and specific drug formulation [9]. In contrast, when delivered intramammarily, cephalosporins have a longer half-life and are predominantly excreted through the urine [10] and udder [11, 12]. A prior study, however, detected cephapirin in the feces (2.12 ± 0.09 µg/kg) up to 6 h after treatment [10], which was also shown for ceftiofur. It was also estimated that 13% of the administered dose of ceftiofur was detected in the feces 5–6 days after IMM treatment [13]. Therefore, understanding the effects of IMM cephalosporin treatment on the fecal microbiota and resistome, or collection of antibiotic resistance genes (ARGs), requires investigation.

Using mathematical modeling, another study predicted that parenteral ceftiofur therapy would reduce the total concentration of *E. coli* in cattle, but would lead to an increase in the fraction of ESBL-resistant *E. coli* [14]. Despite this prediction, several prior studies have not observed a correlation between ceftiofur treatment and an increase in the emergence of ESBL-producing bacterial populations [9, 15, 16]. Although one study of cows receiving systemic ceftiofur treatment in early lactation observed an increase in the abundance of resistant Enterobacteriaceae for 7–8 days, the increase was temporary and was not observed 29–35 days after treatment [17]. Similarly, in feedlot cattle, the combined treatment of chlortetracycline and ceftiofur was linked to an increase in the number of resistant *E. coli* and ESBL and tetracycline resistance genes [18], suggesting co-selection of these ARGs. To further clarify these relationships, we conducted a longitudinal study of dairy cattle to determine how IMM ceftiofur treatment impacts the gut microbiota and abundance of antibiotic resistant bacterial populations through the dry period and early part of lactation.

Since ceftiofur has been detected in the gut when delivered intramammarily [13], we hypothesized that it will select for the growth of specific microbiota members that carry clinically important ARGs. Leveraging metagenomic and bacterial culture techniques, we further hypothesized that IMM ceftiofur treatment will lead to an increase in fecal shedding of ceftiofur-resistant bacteria and cause alterations in the gut microbiome and resistome that are maintained even after antibiotic cessation. Findings from this study could inform decisions about the use of third-generation cephalosporins in livestock, particularly given their importance in human and animal health as well as the global priority to control the emergence of ESBL-producing Enterobacteriaceae on farms.

## Methods

### Study design and sampling scheme

**Fig. 1:**
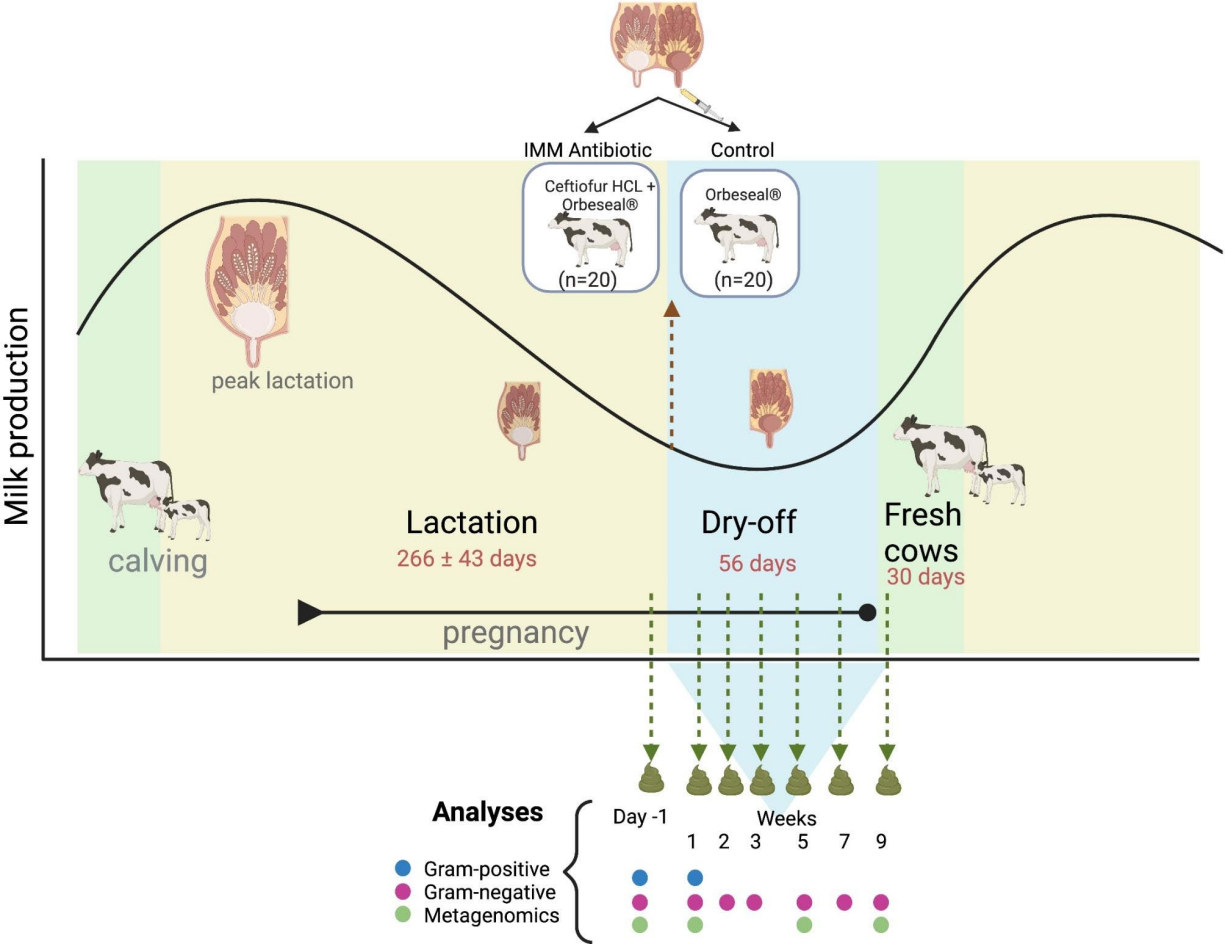
Study design showing the production stage and sampling time points for all 40 animals. Twenty dairy cattle received intramammary ceftiofur (IMM Antibiotic) and 20 matched dairy cattle receiving no IMM antibiotic treatment (Control). Cows were matched based on parity and monthly milk production at Day − 1, which corresponds to the last day of lactation and the day prior to IMM treatment. Matched cows were sampled simultaneously following treatment at weeks 1, 2, 3, 5, and 7 during the dry-off period and again as fresh cows at week 9. Cows remained healthy and were given the same diet. Figure created with BioRender

The aim of this study was to assess the effects of IMM ceftiofur hydrochloride (CHCL) treatment on the gut microbiome of dairy cows at dry-off, the last milking before the dry period (Fig. 1). The study was conducted in 2019 (June-November) at the Dairy Cattle Teaching and Research Center at Michigan State University (MSU), which contained ~ 230 lactating Holstein cows. Forty cows were enrolled at dry-off, with an average of 266 ± 43 days in milk (DIM). Cows were only enrolled if they met the following inclusion criteria: no antibiotic treatment during the last 90 days of lactation and a somatic cell count (SCC) of < 150,000 cells/mL using the most recent Dairy Herd Improvement Association (DHIA) test. The cows were randomly assigned to one of two treatment groups and were matched based on parity and monthly milk production. The antibiotic-treated group (*n* = 20) received 4 IMM infusions (1 per mammary gland) that each contained 500 mg ceftiofur (SpectramastDC®; Zoetis Animal Health) after the last milking plus an internal IMM teat sealant containing bismuth subnitrate (Orbeseal®; Zoetis Animal Health). The control group received only the internal IMM teat sealant without the SpectramastDC®.

Fecal grab samples were collected from each animal’s rectum using clean obstetric sleeves on the last day of lactation, which corresponded to the day prior to IMM treatment (Day − 1). The matched cows were re-sampled simultaneously at weeks 1, 2, 3, 5, and 7 during the dry-off period and again as fresh cows at week 9 (Fig. 1). Samples were collected within 2 days of each other per week and thus, are reported by week in the analysis. This longitudinal sampling scheme was chosen to determine whether any resistant bacterial populations and/or ARGs persisted beyond the ~ 8-week dry-off period and intro the fresh period (week 9) in the treated versus control cows. This timeline also allowed for an assessment of potential impacts due to physiological and dietary changes that occur during the fresh period at week 9. Following collection, each sample was homogenized by hand massage in a whirl-pak bag and immediately aliquoted for bacterial culture and DNA extraction for metagenomic next-generation sequencing (mNGS). For the latter, 0.25 g of feces per sample was preserved at -80ºC in 750 µl of 190 Proof ethanol (95% ethanol) as suggested [19, 20].

Data about ambient temperature and relative humidity were obtained from the East Lansing MSU Horticultural Station through Enviroweather (https://enviroweather.msu.edu/) (Additional file 1, Table S1). Diet ration reports and nutrient amounts given to the cows during the study (one report per lactation phase) were recorded by farm personnel using Spartan Dairy 3 software and were chosen based on recommendations provided by the National Research Council’s 2001 Nutrient Requirements of Dairy Cattle (NRC 01) [21] (Additional file 1, Table S2). Animals from both treatment groups were given the same diet at each sampling, which corresponded to their physiological and productive stage at the time. Moreover, the same farm personnel evaluated animal health status over the course of the study and all cows remained healthy. Researchers were blinded to treatment status during sample collection and the subsequent laboratory analyses.

### Sample size justification

The sample size was determined using the ‘pwr.t.test()’ function from the ‘pwr’ package (version 1.3-0) in R. This computation incorporated a power of 80% and a significance level of 0.05, catering to a two-sided alternative hypothesis for a paired investigation. An assumed moderate effect size, quantified using Cohen’s d, was set at 0.46. Given these conditions, 40 cows, equally divided into a group of 20 for treatment and a group of 20 for controls, were deemed necessary to detect differences in the abundance of ARGs, particularly those encoding β-lactamases, and taxa between the two groups. This sample size also respects ethical principles by minimizing the use of animals while still ensuring reliable results. Furthermore, it is in accordance with logistical considerations and resource availability, reinforcing its appropriateness for the scope and objectives of the study.

### Quantification of antibiotic-resistant bacteria

Total bacterial counts were quantified and presented as colony-forming units (CFUs) per gram (g) of feces. Moreover, the percentage of ceftiofur- and ampicillin-resistance was quantified for Gram-positive bacteria on day − 1 and week 1 as well as Gram-negative bacteria on day-1 through week 9. Fecal samples were diluted at a concentration of 10^− 1^ using 1 g of feces and 9 ml of 1× PBS and plated in duplicate on selective media using a spiral autoplater (Neutec Group Inc.). The media for Gram-negative bacteria was MacConkey lactose agar (MAC; Criterion®), whereas Columbia Nalidixic Acid agar (CNA; BD Difco ®) with 5% sheep blood was used for Gram-positive bacteria. Amphotericin B (4 µg/ml) was also added to inhibit fungal growth along with varying concentrations of antibiotics. To recover resistant Gram-negative and Gram-positive bacteria, a ceftiofur (Cef) concentration of 8 µg/ml was used per the Clinical and Laboratory Standards Institute (CLSI) guidelines VTE01 for animal isolates using values for Enterobacteriaceae (Gram-negative) and *Staphylococcus* spp. and *Enterococcus* spp. (Gram-positive) [22]. These values were chosen because guidelines are not available for human isolates. Comparatively, 25 µg/ml of ampicillin (Amp) was used to recover resistant Gram-positive bacteria [23], while 32 µg/ml of Amp was used for resistant Gram-negative bacteria based on the M100 CLSI guidelines for human isolates of *Enterococcus* spp. and Enterobacteriaceae, respectively.

The antibiotic concentration on MAC that inhibited susceptible (S) bacteria and enabled the growth of resistant (R) strains was tested with the following control strains: *E. coli* ATCC 25,922 (Amp^S^, Cef^S^), *E. coli* ATCC 35,218 (Amp^R^, Cef^S^), and three ESBL-producing *E. coli* strains (Amp^R^, Cef^R^) obtained from clinical samples in a prior study [24]. CNA controls included ATCC 29,212 (Amp^S^, Cef^R^), ATCC 29,213 (Amp^S^, Cef^S^), *Listeria monocytogenes* ATCC 3382 (Amp^S^, Cef^R^), *L. monocytogenes* ATCC 19,115 (Amp^S^, Cef^R^), *Streptococcus pneumoniae* ATCC 49,619 (Amp^S^, Cef^S^), *Streptococcus equi* subsp. *zooepidemicus* ATCC 700,400 (Amp^S^, Cef^S^), and *Streptococcus agalactiae* strain COH1 (Amp^S^, Cef^S^). Inhibition of Gram-negative bacteria was tested with *E. coli* ATCC 25,922 and the ESBL-producing *E. coli* strains.

**Fig. 2:**
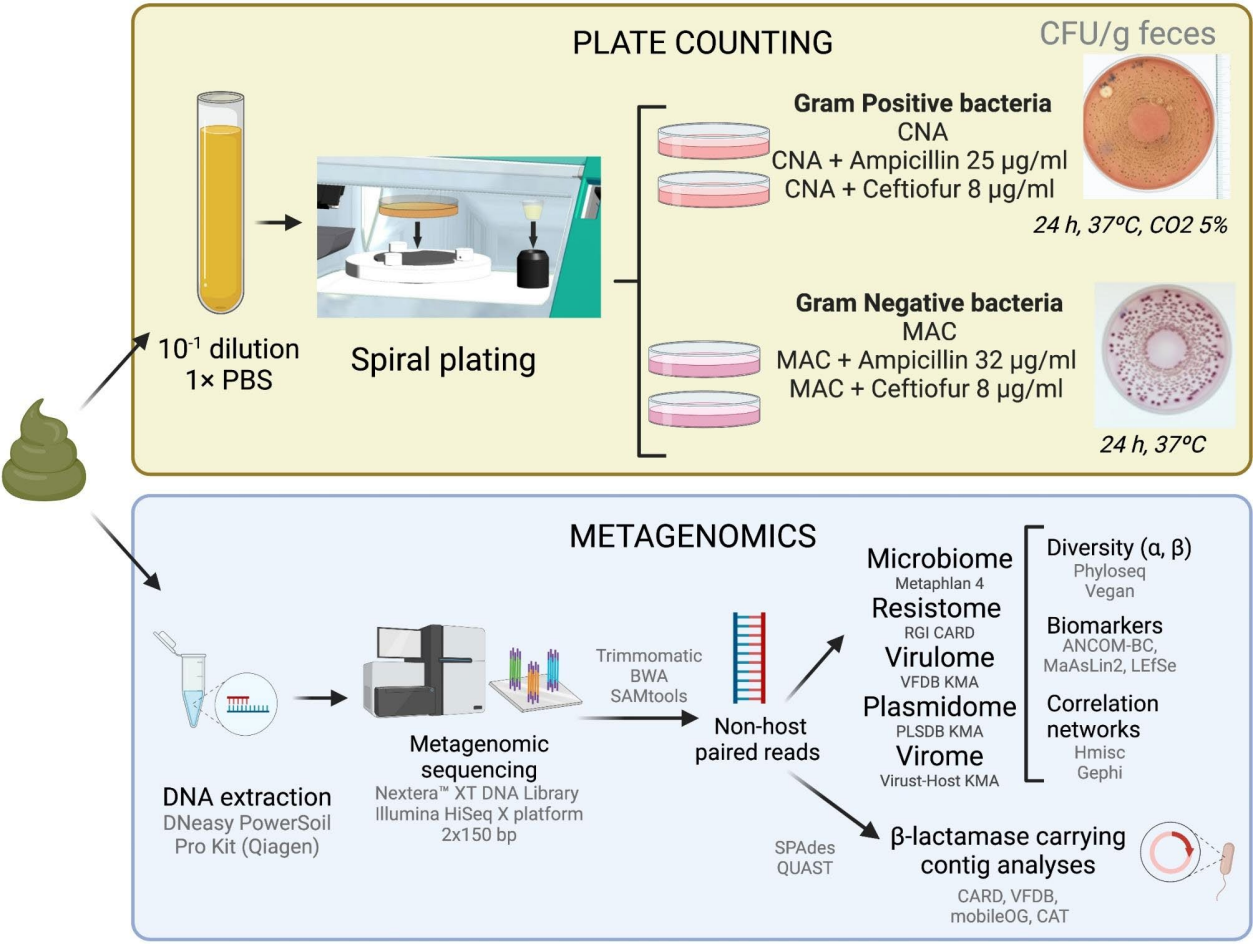
Summary of methods used for the quantification of Gram-positive and Gram-negative bacteria and metagenomics. The goal of these analyses was to identify the effects of intramammary (IMM) ceftiofur treatment on the cattle fecal microbiome using both culture-based methods and sequencing. Figure created with BioRender.com

To culture the bacteria using the spiral autoplater, different modes and volumes of the diluted sample were used. Specifically, 40 µl (C mode) was used for MAC, 100 µl (C mode) for MAC with ampicillin, and 400 µl for MAC with ceftiofur. Additionally, for CNA, 40 µl (C mode) was used, while CNA with ampicillin and CNA with ceftiofur required 200 µl (linear mode) and 100 µl (C mode), respectively. The plates were incubated at 37^o^C for 24 h under aerobic conditions (MAC) or in the presence of 5% carbon dioxide (CNA) (Fig. 2). Media controls were also plated to test each batch of MAC for the ability to inhibit Gram-positive bacteria using *Staphylococcus aureus* ATCC 29,213 and *Enterococcus faecalis* ATCC 29,212. Lastly, Gram-negative ceftiofur-resistant colonies recovered from the plates were characterized using oxidase tests (OxiStrips^™^, Hardy Diagnostics) and Chromocult® Coliform agar (Merck KGaA, Darmstadt, Germany) to test for β-glucuronidase and β-galactosidase activity.

### Statistical analysis of bacterial counts

Data analysis and plot generation were performed using R v.4.2.2 and RStudio v.2023.03.0 + 386. To ensure a more suitable scale for analyzing bacterial counts, the CFU/g values were transformed to log_10_ values. This transformation involved adding 1 to the raw counts to account for instances of 0 counts, as the log_10_ of 1 is 0. The R base function log10() was then applied to obtain the log10-transformed CFU/g values. The rstatix package v.0.7.2 was used to generate summary statistics, including measures of central tendency, through the ‘get_summary_stats()’ function.

The Shapiro-Wilk test was performed using the rstatix R function ‘shapiro_test()’ to determine whether the count data were normally distributed; a resultant *p*-value below 0.001 was indicative of a non-normal distribution. Accordingly, the non-parametric one-sided paired Wilcoxon signed rank test was used with the ‘wilcox_test()’ function from the rstatix package to detect differences between the ceftiofur-treated and control animals at a single time point or between the same group at 2 time points. This test represents an appropriate alternative when the assumption of normality, which is inherent to parametric methods, is violated. To account for repeated measures in either the ceftiofur-treated or control animals over the 9-week sampling period (7 samples/cow), the unpaired Friedman’s rank-sum test was used. This test represents a non-parametric alternative to the one-way repeated measures ANOVA and was performed with the ‘friedman_test()’ function from the rstatix package. Both tests were used to compare the number of CFU/g as well as the proportion of resistant bacteria between treatment groups and time points. A *p*-value of ≤ 0.05 was indicative of a statistically significant difference.

The ‘ggline()’ function from the ggpubr package v.0.6.0 was used to create line plots, with the ‘geom_point()’ function from the ggplot2 package v.3.4.2 used to add points. To display significant differences, *p*-values were incorporated into the plots using “geom_signif()” and “stat_pvalue_manual()” functions.

The R package Aligned Rank Transform (ARTool) v.0.11 was used with the art() function to analyze how multiple factors, including physiology, diet, weather, and time post-treatment can affect the log_10_ of CFU/g. The fixed effects in the model were lactation phase, time point, group, ambient temperature, and diet type, while the cow ID was considered a random effect. After creating the model, the summary() function was used to generate a comprehensive summary of the model. Finally, the Analysis of Deviance Table (Type III Wald F tests with Kenward-Roger df) was obtained with the anova() function, which provided the significance of each term in the model by considering its contribution after accounting for the other terms.

### DNA isolation and metagenomic next generation sequencing (mNGS)

Fecal DNA from samples collected on day − 1 and weeks 1, 5, and 9, were selected for DNA extraction and sequencing. The samples were centrifuged at 16,000 rpm for 5 min at 4 °C to remove the supernatant and residual ethanol, which was followed by two washes with 1 ml of molecular grade 1× PBS that was removed as done in the prior step. The DNeasy PowerSoil Pro Kit (Qiagen, Germantown, MD, USA) was used to extract DNA from each sample according to the manufacturer’s instruction followed by two wash steps using the C5 solution to improve the DNA quality ratio prior to DNA elution. Genomic DNA was measured using a Qubit 3.0 with the dsDNA High Sensitivity (HS) assay kit (Invitrogen™, MA, USA), while the quality ratios 260/230 and 260/280 were quantified with a NanoDrop ND-1000 (Thermo Fisher Scientific Inc, DE, USA). DNA extractions were performed in 13 batches by one individual using the same protocol.

The metagenomic composition of cattle feces was analyzed for a total of 159 samples collected one day prior to treatment (day − 1) and at weeks 1, 5, and 9 post-treatment. The DNA extracted from each sample (average: 1277.3 ng ± 310.5 ng of dsDNA) was sent to CosmosID (Rockville, MD, USA) for mNGS. Libraries were prepared with the Nextera™ XT DNA Library Preparation Kit (Illumina, San Diego, CA, USA) and sequenced on the Illumina HiSeq X platform 2 × 150 bp (Fig. 2). The DNA library preparations and sequencing were performed in a single batch, except for one sample that was re-sequenced because of quality issues.

### Sequence processing

Paired-raw sequences were first analyzed with FastQC v. 0.11.7 [25] to assess quality and MultiQC v.1.7 [26] was used to summarize FastQC results into a single report. Trimommatic v.0.39 [27] was applied to remove low-quality reads and adapters used for Illumina sequencing with the parameters ILLUMINACLIP:nextera.fa:2:30:10:3:TRUE, LEADING:10, TRAILING:3, SLIDINGWINDOW:4:15 and MINLEN:36. Burrows-Wheeler Aligner (BWA) v.0.7.15 [28] and SAMtools v.1.4.1 [29] were used to remove bovine DNA reads (*Bos taurus*, ARS-UCD1.2) and BEDTools v.2.30.0 was used to convert non-host reads from BAM format to FASTQ.

### Microbiota characterization

Non-host paired reads were analyzed using the Metaphlan 4 software [30] and the mpa_vJan21_CHOCOPhlAnSGB_202103 database to identify taxonomic features. The minimum read length was set to 60 bp, and the minimum mapping quality value was set to -1. The robust average quantile value was set to 0.1, and the Bowtie2 presets were set to very-sensitive-local. The normalized abundance score for each taxonomic feature was calculated by dividing the number of reads by the number of genome equivalents, which were determined by dividing the total number of sequenced base pairs by the estimated average genome size using the MicrobeCensus v.1.1.1 method [31].

### Resistome profiling

To characterize the resistome, the Resistance Gene Identifier (RGI) v.6.0.0 software [32] was used to analyze non-host paired metagenomic reads based on homology models. The Comprehensive Antibiotic Resistance Database (CARD) v.3.2.5 was aligned with the RGI software for metagenomic short reads with the command ‘rgi bwt’ using K-mer Alignment (KMA) v.1.4.9 with 20 bp k-mers as seeds while setting the coverage and identity to at least 50% and 80%, respectively. These settings, especially the relatively low coverage parameter, were chosen to account for the shallow sequencing depth encountered in the study. Moreover, the settings specified the use of each query sequence to match only one template and results were reported at the drug class and allele levels. Resistance determinants based on point mutation (SNP) models, such as those identified with the rRNA, protein variant, and protein overexpression models were excluded. The abundance of each ARG allele was normalized by dividing the depth by the number of genome equivalents and multiplying the result by 100. A heatmap containing the 70 most abundant ARGSs was made with the R package pheatmap v.1.0.12. The heatmap values were modified to represent the log_10_ of the normalized abundance. A small increment, specifically ‘0.0000001’, was added to each value to counteract the computational issues associated with a logarithm of zero. Hierarchical clustering was performed on the samples and genes using the Ward D2 method.

### Identification of plasmids, virulence factors and viruses

To identify plasmids, virulence factors, and virus sequences, the Plasmid Sequence Database (PLSDB) (updated on 06-23-2020) [33], Virulence Factor Database (VFDB) setB (12-08-2022) [34], and Virus-Host (11-29-2022) [35] nucleotide databases were utilized, respectively (Fig. 2). KMA v.1.4.3 [36] was employed with 20 bp k-mers, requiring each query sequence to match only one template. Normalization was conducted as described for the resistome.

### Diversity analyses of the microbiome and resistome

The R package Phyloseq v.1.38 [37] was used to analyze alpha and beta diversity of the microbiome (i.e., microbiota, plasmids, virulence genes, and viruses) and resistome profiles. The alpha diversity was calculated using the number of reads for microbiota or the depth for features identified with KMA and measured with the Shannon index and Observed (richness) index. Normalized abundances were used to calculate the beta diversity based on Bray-Curtis dissimilarities with the Vegan package v.2.6-4 [38].

### Statistical analysis of microbiome features

Microbiome and resistome normalized abundances and alpha diversity indexes were analyzed with the Shapiro-Wilk’s test, which indicated a non-normal distribution of the data (Shapiro-Wilk, *P* < 0.05). Consequently, paired and one-sided Wilcoxon signed rank tests were used to compare treatment groups per time point. The unpaired Friedman’s rank-sum test was used to test for time-based fluctuations, which accounts for repeated measures and was described for bacterial counts. Permutational multivariate analysis of variance (PERMANOVA) with 999 permutations and principal coordinate analyses (PcoA) were performed to compare the beta diversity between treatments and time points with the Vegan function adonis2 [38]. Mixed-effects models were applied to identify factors associated with variations in microbiota abundance across time points using ARTool as described for Gram-negative bacteria; the same variables were examined.

### Biomarker identification

The analysis of differentially abundant features was carried out with three different approaches: 1) Linear Discriminant Analysis (LDA) Effect Size (LefSe), which identifies the effect relevance of a differential feature based on an algorithm that includes non-parametric tests and LDA [39]; 2) Analysis of compositions of microbiomes with bias correction (ANCOM-BC), which uses linear regression models and corrects for bias induced by sample differences [40]; and 3) Microbiome Multivariable Associations with Linear Models (MaAsLin2) [41] that uses generalized linear and mixed models. A consensus approach was used to ensure robust identification of differentially abundant features; only differentially abundant features (*P* < 0.05) identified with two or more pipelines were reported. To generate plots for the biomarkers, the abundance scores for each feature, whether gene or taxa, were normalized by dividing by the average score for the corresponding feature at each time point. This provided a fold-change (FC) value for each feature. Subsequently, bar plots were constructed to indicate the group mean FC along with the corresponding standard error at each time point. The function ‘ggbarplot()’ from the ggpubr package v.0.6.0 was employed to create these visualizations, with the standard error displayed.

### Characterization of β-lactamase carrying contigs

Non-host paired metagenomic sequences were assembled using SPAdes v.3.13.0 with the option ‘--meta’ [42] and MEGAHIT v.1.2.9 with the option ‘meta-large’. Assemblies obtained with both tools were evaluated with Quast v.5.0.0 using the script ‘metaquast.py’ with a custom reference list of the 20 most abundant microbial species [43]. MultiQC v.1.7 was used to consolidate individual Quast reports. Key parameters including N50, L50, the largest contig size, total length, misassembles, mismatches, indels, and the genome fraction were compared between the SPAdes and MEGAHIT assembly methods. SPAdes was superior, delivering significantly longer total lengths (Wilcoxon signed rank test, *P* = 0.00013), and registering fewer misassembles, mismatches, and indels (Wilcoxon signed rank test, *P* < 0.05). Consequently, assemblies generated by SPAdes were used for downstream analyses. The proportion of reads mapping a contig was identified with BWA v.0.7.15 [28] and SAMtools v.1.4.1 [29]. Prodigal v.2.6.3 (PROkaryotic Dynamic programming Gene-finding Algorithm) [44] was used to translate contigs into amino acid sequences. Open-reading frames (ORFs) obtained with Prodigal were mapped to the protein databases CARD [32], VFDB [34], and mobile orthologous groups database (mobileOG-db) [45] using DIAMOND v.2.0.1 and the blastp command [46]; the minimum sequence identity was set to 80% and the e-value at 0.001. Contigs carrying β-lactamases that confer resistance to cephalosporines with a length ≥ 500 bp were extracted with seqtk and taxonomically classified using the contig annotation tool (CAT) v.5.2.3 with the database 2021-01-07_CAT [47].

### Network analysis

Correlations between ARGs, plasmids, viruses, virulence factors, and bacterial genera were identified by calculating Spearman’s correlation coefficients; only coefficients (ρ) greater than 0.75 and p-values < 0.01 were included in the networks. Significant correlations were analyzed with the R package Hmisc v.4.7-2 [48]. Network plots and statistics were analyzed with Gephi v.0.9.2 [49], including the degree and betweenness centrality. The comparisons of centrality measures among β-lactam ARGs were analyzed between treatment groups and time points using one-sided and paired Wilcoxon signed rank tests.

## Results

### Characteristics of the study population and sampling scheme

In this longitudinal study, 40 Holstein cows were enrolled at the end of lactation. Animals were matched based on parity and monthly milk production and pairs were randomly assigned to the treatment or control group. Importantly, no difference in the average DIM was observed between the antibiotic-treated (*mean* = 262.69 ± 37) and control (*mean* = 269.59 ± 47) cows (Wilcoxon signed rank test, *P* = 0.22). Mastitis was ruled out in these cows as the somatic cell counts (SCC) in milk was an average of 34,8718 ± 23,602 cells/mL (antibiotic group *mean* = 35,300 cells/mL; control group *mean* = 34,4211 cells/mL). Fecal samples were collected from all animals through the 9-week period except for one cow in the antibiotic group. This cow had a C-section in week 9 and hence, a final sample was not obtained. Sampling began in the summer and ended in the fall and hence, the temperatures gradually decreased over the course of the study (Additional file 1, Table S1). Moreover, all cows received four diets that corresponded to their lactation phase, which included the maintenance (day − 1), dry (weeks 1–5), close-up (week 7), and fresh (week 9) diets (Additional file 1, Table S2). All cows were pregnant and healthy during the study with most giving birth around the ninth week after dry-cow therapy.

### Bacterial quantities vary across samples and groups

**Fig. 3:**
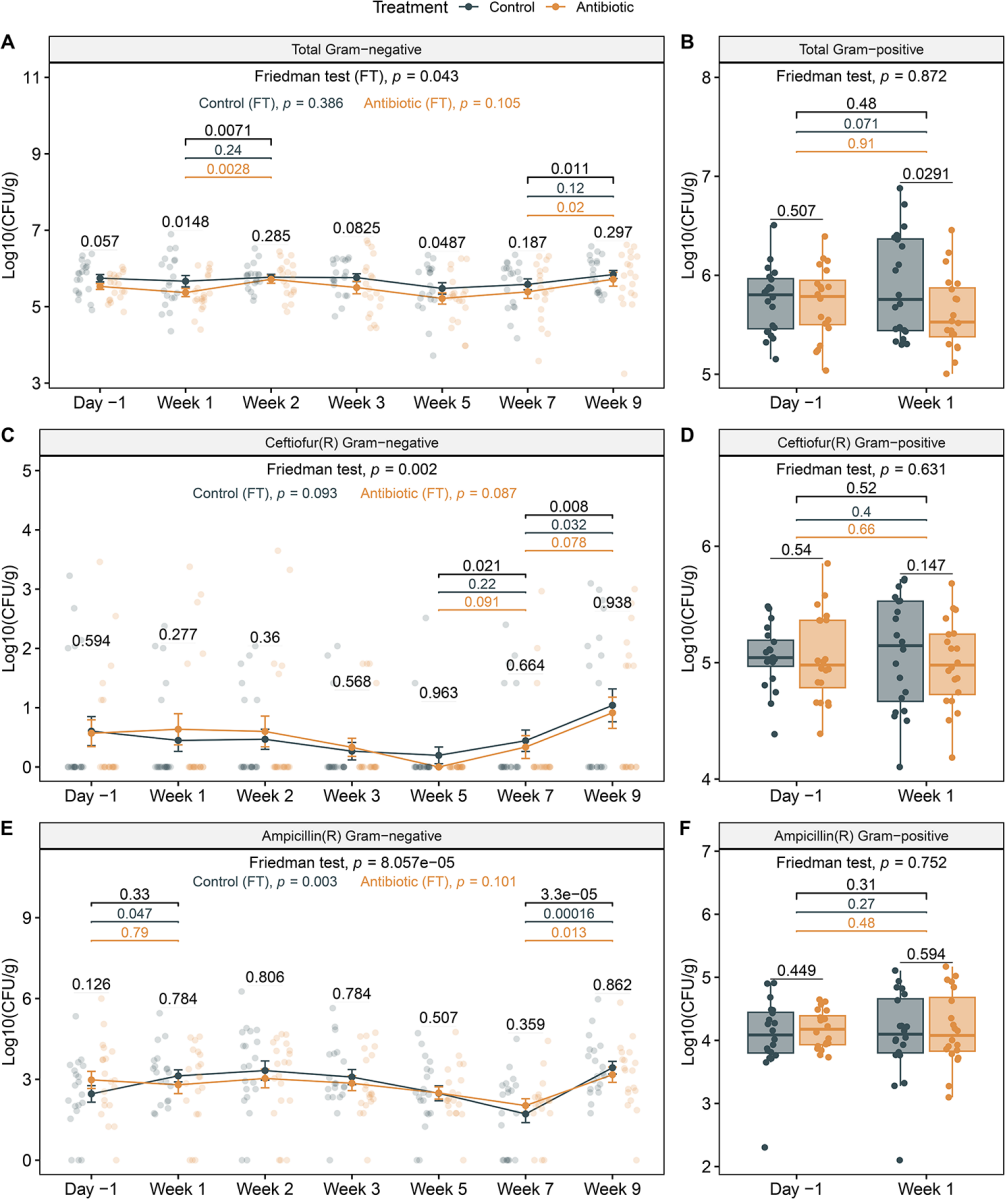
Number of bacterial colony-forming units (CFUs) per gram of feces. Total number (log_10_ CFUs/g) of **A**) Gram-negative and **B**) Gram-positive bacteria recovered from each sample in the ceftiofur-treated (orange) and control (gray) cows. Numbers of ceftiofur-resistant **C**) Gram-negative and **D**) Gram-positive bacteria are also shown along with the number of ampicillin-resistant **E**) Gram-negative and **F**) Gram-positive bacteria. Numbers are plotted before (Day − 1) and after treatment for Gram negative bacteria through 9 weeks and after 1 week for Gram-positive bacteria. Line plots show means and standard error bars with sample counts represented as dots. Boxplots indicate the median, lower, and upper quartiles, and the whiskers represent extreme values in the distribution. *P*-values were calculated using a paired Wilcoxon signed rank test to compare treatment groups within a sampling point and the same group across two samplings. The per animal variability over time was calculated with the Friedman rank-sum test (FT), which accounts for repeated measures and is shown per treatment group for the Gram-negative bacteria. Significant *p*-values between sampling points are represented for all cows (black) as well as control (grey) and antibiotic-treated (orange) cows

Slight variation in total bacterial counts was observed at different time points throughout the sampling period. At week one, the antibiotic-treated cows had significantly lower Gram-negative bacterial counts (Wilcoxon signed rank test, *P* = 0.0148), which was also observed at week five (Wilcoxon signed rank test, *P* = 0.0487; Fig. 3A). No difference in total Gram-negative bacterial counts was observed between groups at weeks 2, 3, 7, or 9 (Wilcoxon signed rank test, *P* > 0.05). For the Gram-positive bacteria, significantly higher counts were recovered in the control animals relative to the ceftiofur-treated animals one week after treatment (Wilcoxon signed rank test, *P* = 0.029; Fig. 3B).

Despite the slightly lower abundance of Gram-negative CFUs one day prior to treatment (day − 1) in the antibiotic-treated (*mean* = 4.41 × 10^5^ CFUs) cows compared to the control cows (*mean* = 8.25 × 10^5^ CFUs), the difference was not significant (Wilcoxon signed rank test, *P* = 0.057). To ensure that this difference did not impact the results, however, we also calculated the fold-change by dividing the logarithm with base 10 (log_10_) of the CFU/g at each time point by the log_10_ of the CFU/g at day − 1 for each animal. Using this approach, no significant differences were detected in the total Gram-negative bacterial counts between the ceftiofur-treated and control groups at any of the samplings (Wilcoxon signed rank test, *P* > 0.05). Bacterial count data are provided in Additional file 1, Table S3. Moreover, use of mixed-effects models demonstrated that the total number of Gram-negative bacteria was not influenced by treatment (ANOVA, *P* = 0.14) but by the interaction of lactation phase and ambient temperature (ANOVA, *P* = 0.001), which was similar to the interaction between time post-treatment and temperature (ANOVA, *P* = 0.002). Warmer temperatures and the period near the end of lactation (prior to dry-off) were associated with greater counts of Gram-negative bacteria.

### The level of phenotypic resistance varies across samples and groups

The percent of Gram-negative and Gram-positive bacterial populations with phenotypic resistance to ampicillin and ceftiofur was determined for all samples. Overall, 90% of the samples had Gram-negative bacteria with resistance to ampicillin, while 24% of the samples had Gram-negative bacteria with resistance to ceftiofur. Regardless of treatment status, a greater proportion of the total number of Gram-negative bacteria had resistance to ampicillin (2.76%$\pm$10.60%) than resistance to ceftiofur (0.02%$\pm$0.09%) within a fecal sample (Additional file 2, Figures S1A and S1C). By contrast, Gram-positive bacteria with resistance to both ampicillin and ceftiofur were recovered from all (100%) of the samples from both the antibiotic-treated and control animals. For these Gram-positive bacteria, a greater proportion of the total number had ceftiofur resistance (28.16% ±21.82%) than ampicillin resistance (4.81% ± 6.06%) per sample (Additional file 2, Figures S1B and S1D). A difference between treatment groups was only observed for the percentage of ampicillin-resistant Gram-positive bacteria, which was higher in the ceftiofur-treated cows than the control cows at week 1 (Wilcoxon signed rank, *P* = 0.0413).

Similar results were obtained when the total number of resistant bacteria (CFUs/g) was examined, which did not take the total bacterial counts into consideration. Although no differences were identified between the number of resistant Gram-negative bacteria in the treated and control groups for either antibiotic within a sampling, some notable differences were still observed. For example, a subset (25%) of the treated cows shed higher levels of ceftiofur-resistant bacteria for up to 2 weeks post-treatment when compared to the control cows (Fig. 3C). In addition, the total number of ceftiofur-resistant Gram-negative bacteria increased in both groups at week 9 relative to week 7, though this increase was only significant for the control cows (Wilcoxon signed rank test, *P* = 0.032). Regardless, a gradual increase in ceftiofur-resistant bacteria was observed for both groups by week 9 as compared to week 5 (Wilcoxon signed rank test, *P* < 0.02) while the number of ampicillin-resistant Gram-negative bacteria increased significantly in both groups between weeks 7 and 9 (Wilcoxon signed rank test, *P* < 0.01; Fig. 3E). No differences were observed for the number of resistant Gram-positive bacteria by group or between the two samplings (Fig. 3D F). Statistical comparisons between groups and within groups are provided in Additional file 1, Tables S4 and S5.

### Metagenomic sequencing metrics

The average number of reads (151 bp) per sample was 5.74 (± 1.1) million, and no difference was observed in this number between treatment groups (Wilcoxon signed rank test, *P* = 0.11) (Table 1). In week 5, however, samples from the antibiotic-treated group had a lower number of reads compared to controls (Wilcoxon signed rank test, *P* = 0.035) (Additional file 2, Figure S2). The mean proportion of duplicate sequences was 11.36% (± 2.25), while the GC content was 48.00% (± 0.67) and 9.23% (± 1.41) had failed sequences.

**Table 1:** Metagenomic sequencing metrics from cattle fecal DNA.

Feature	Mean	p-value*				
		Lactation Phase	Temperature	Treatment	Time post-treatment	Lactation Phase: Temperature: Treatment
***Reads***						
Raw reads (150 bp)	5,735,481	0.94	0.70	0.84	0.40	0.61
Non-host reads (150 bp)	4,507,080	0.79	0.72	0.73	0.36	0.42
Proportion of host reads (*Bos Taurus %*)	21.417576	**< 0.0001**	0.60	0.83	0.78	0.46
***Proportion in non-host reads***						
Bacteria (%)	11.027	**< 0.0001**	0.12	0.92	**0.05**	0.70
Archaea (%)	0.746	**< 0.0001**	0.89	0.53	0.08	
Eukaryota (%)	0.170					
Viruses (%)	0.004	0.45	0.94	0.33	0.65	0.31
Genome equivalents (Nº)	239.930	0.06	0.47	0.77	0.51	0.10
Average genome size (bp)	2,754,869	**< 0.0001**	0.97	0.33	**< 0.0001**	0.73
***Contig assemblies***						
Contigs (Nº)	341430.855	1.00	0.62	0.99	0.09	0.31
Reads mapping contigs (%)	36.011	**0.01**	0.69	0.37	**< 0.0001**	**0.02**
N50 (Kbp)	0.764	0.98	0.35	0.87	0.88	0.23
L50 (Kbp)	5.703	0.92	0.81	0.63	**0.01**	0.12
Largest contig (Kbp)	38.065	0.15	0.35	0.75	**0.02**	0.38
Length (Mbp)	14.887	0.87	0.72	0.56	**0.01**	0.29

*P-values were calculated with ANOVA using mixed effects models including differences in lactation phase, ambient temperature (ºC), treatment group, time post-treatment, and diet while controlling by cow ID. It should be noted that the factor of diet did not contribute significantly to the model due to the correlation between diet variation and each specific lactation phase. Significant *p*-values are in bold. The analysis could not incorporate the factor of Eukaryota as it was not detected consistently across all samples

After quality control, approximately 4,211.06 (± 763.37) sequences were dropped per sample, corresponding to 0.07% (± 0.01) of the raw reads. On average, 21.42% of the reads corresponded to bovine DNA. No differences were identified between treatments in the number of non-host paired reads (Wilcoxon signed rank test, *P* = 0.129). However, cows treated with ceftiofur had a significantly lower number of non-host reads in week 5 (Wilcoxon signed rank test, *P* = 0.041), but not in the number of genome equivalents (Wilcoxon signed rank test, *P* = 0.062) (Additional file 2, Figure S2). The proportion of microbial taxa identified with Metaphlan 4 corresponded to 11.95% of the non-host reads, which varied significantly over time showing lower abundance in samples taken during the dry-off (weeks 1–5) and fresh (week 9) periods as compared to late lactation (day − 1). The lactation phase significantly impacted the proportion of host reads, average genome size, and reads mapping to contigs. Similarly, time post-treatment influenced the metrics of contig assembly (Table 1).

### Taxonomic profiling reveals differences across lactation phases

**Fig. 4:**
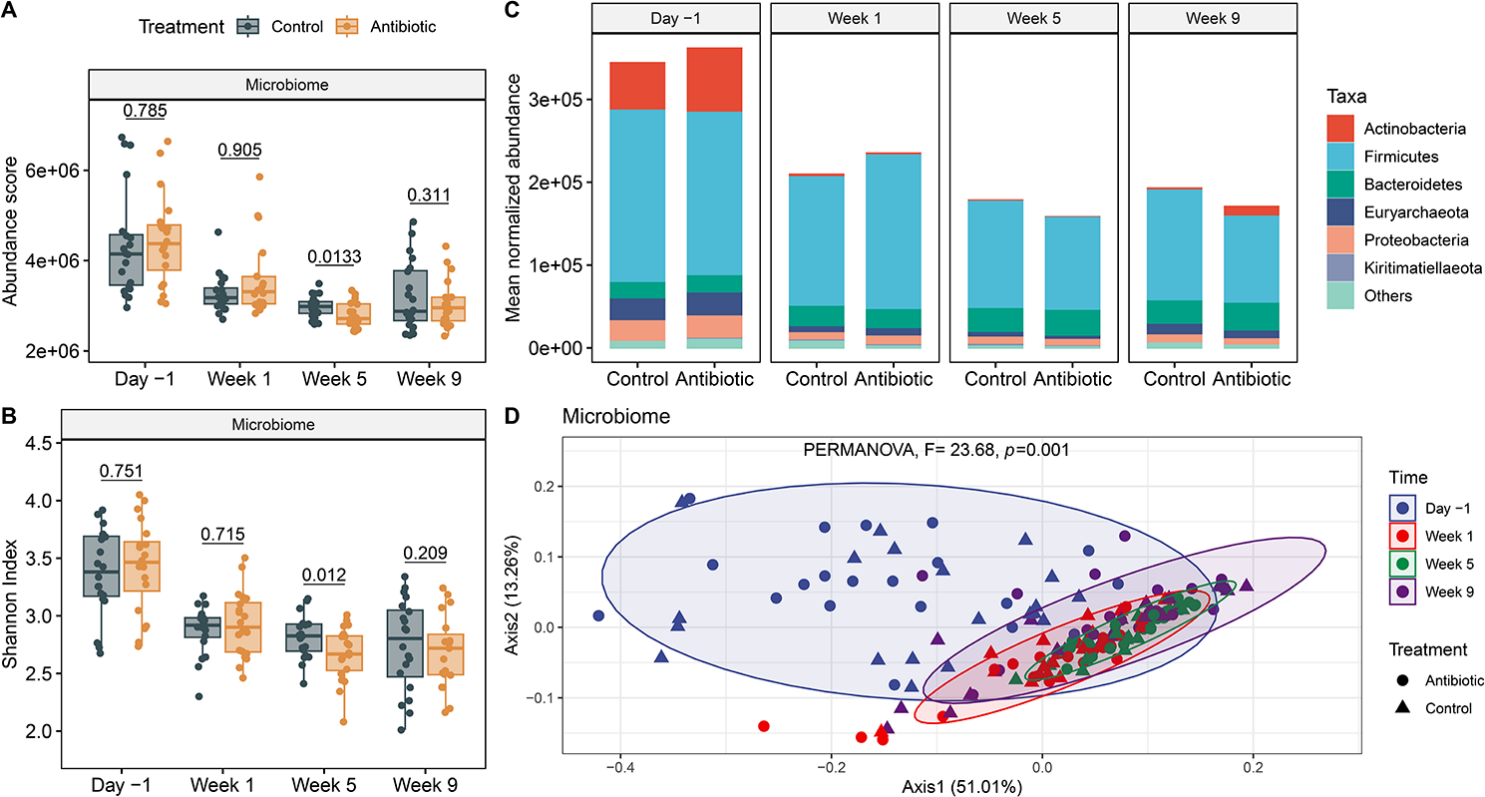
Microbiota diversity and composition before (Day − 1) and 1, 5, and 9 weeks after dry-cow therapy. **(A)** Normalized abundance and **(B)** Shannon Index (alpha diversity) among ceftiofur-treated (orange) and control (grey) cows. Each boxplot shows the median, lower, and upper quartiles with the whiskers representing extreme values in the distribution. **(C)** The mean normalized abundance of microbial taxa at the phylum level, and **(D)** a PCoA of the Bray-Curtis dissimilarity. Ellipses in the PCoA are clustered by sampling point and contain at least 90% of the samples. *P*-values were calculated using a paired Wilcoxon signed rank test to compare treatment groups within a sampling point

The microbiome was dominated by bacteria (92.29%), archaea (6.25%), eukaryotes (1.42%), and viruses (0.03%). The normalized abundance of microorganisms was significantly higher during the late lactation period (Day − 1) compared to the dry-off and pre-calving periods (Wilcoxon signed rank test, *P* < 0.001) (Fig. 4A). Mixed effects models were utilized to determine the contributing factors of microbial abundance fluctuations. In this analysis, lactation phase (ANOVA, *P* = 0.0002) was associated with the observed differences in microbial abundance, while environmental temperature and treatment group did not significantly impact the taxonomic abundance (ANOVA, *P* = 0.12, *P* = 0.92, respectively). Differences in alpha (Shannon) diversity were observed over the sampling period (Friedman’s test, *P* = 2.77e-10). The most diverse communities were detected before dry-cow therapy (Day − 1) (Fig. 4B). When comparing antibiotic-treated versus control cows, the abundance and alpha diversity of taxa were only significantly lower in week 5 (Wilcoxon signed rank test, *P* = 0.01).

Significant changes in the bacterial composition were also detected across samplings as visualized in a relative abundance plot (Fig. 4C) and a Bray-Curtis dissimilarity ordination (PERMANOVA, *F* = 23.68, *R*^*2*^ = 0.31, *P* = 0.001) (Fig. 4D). The day before dry-off (day − 1) was characterized by a higher abundance of Actinobacteria, Firmicutes, Euryarchaeota and Proteobacteria compared to weeks 1, 5, and 9. Stratifying by treatment status detected several differences in the abundance of various taxonomic groups (Additional file 1, Table S6). Cows treated with ceftiofur, for instance, had a higher abundance of Ruminococcacea and a lower abundance of *Romboutsia* and Rickenellaceae one week after treatment, compared to the control group (Wilcoxon signed rank test, *P* < 0.05) (Additional file 2, Figure S3). At week 5, however, a lower abundance of several taxa including families Ruminococcacea, Lachnospiraceae, and Methanobacteriaceae, were detected in the antibiotic-treated cows.

Although a slight rebound in the Actinobacteria population was observed at week 9, this was only observed in the antibiotic-treated group (Fig. 4C, Figure S3). *Campylobacter* were also more abundant in the ceftiofur-treated cows compared to the controls. Overall, the differences in the taxonomic profiles between the treatment groups demonstrated a persistent effect of the antibiotic on certain bacterial groups, but not on the overall microbiota composition. No taxa were consistently affected over the 9-week period, though one taxon was persistently affected in weeks 1 and 9, three taxa in weeks 1 and 5, and five in weeks 5 and 9. Given that differences were observed due to time post-treatment, which included transitions in diet, temperature decreases, and changes in pregnancy stage, we only compared between treatment groups within each time point for the microbial abundance and diversity metrics.

### Resistome composition analyses identified persistent ARG signatures

**Fig. 5:**
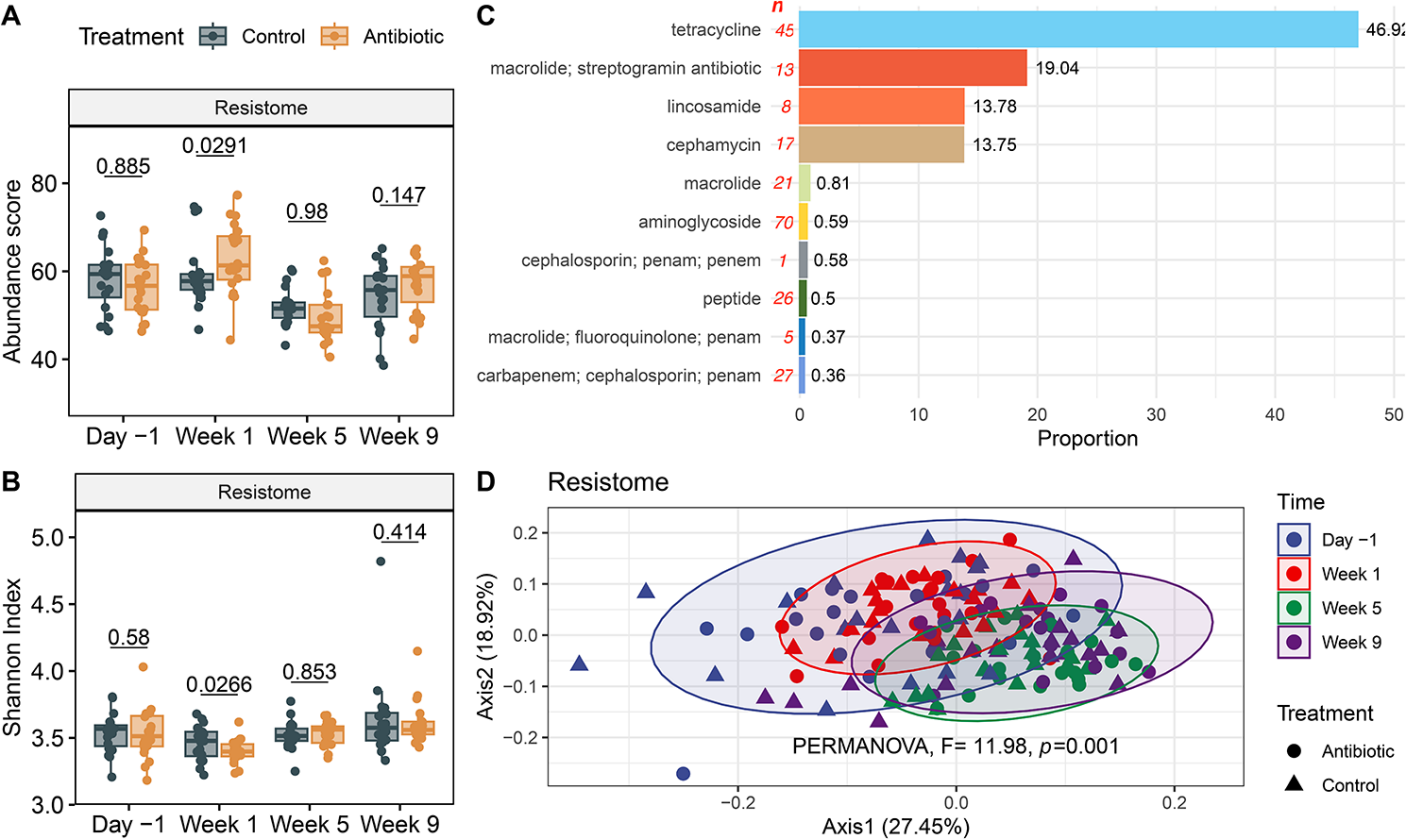
Fecal resistome composition of dairy cows during the 9-week study. **A**) The normalized abundance score and **B**) Shannon Index comparing antibiotic-treated (orange) and control (grey) animals is shown. Each boxplot includes the median, lower, and upper quartiles with the whiskers representing extreme values in the distribution. **C**) The resistome composition at the drug class level shows the average proportion; “n” indicates the number of genes assigned to a given class. **D**) PCoAs of the Bray-Curtis dissimilarity clustered by sampling point (ellipses contain at least 90% of the samples). *P*-values were calculated using a paired Wilcoxon signed rank test to compare treatment groups within a sampling point

After treatment with ceftiofur, a significantly higher abundance of ARGs was observed in animals only at week 1 (Wilcoxon signed rank test, *P* = 0.03) (Fig. 5A) along with a lower Shannon index (Wilcoxon signed rank test, *P* = 0.03) (Fig. 5B). Similarly, the number of observed ARG alleles was lower in week 5 in cows treated with ceftiofur (Wilcoxon signed rank test, *P* = 0.04). The main ARG drug classes identified were for resistance to tetracyclines (46.92%), macrolides and streptogramins (19.04%), lincosamides (13.78%), and cephamycins (13.75%) (Fig. 5C). The mean normalized allelic composition of ARGs varied significantly over time (PERMANOVA, *F* = 11.98, *R*^*2*^ = 0.19, *P* = 0.001), although samples from different time points generally overlapped in the PCoA (Fig. 5D). At the gene level, the tetracyline resistance genes, *tet(W)*, *tet(Q)* and *tet(O)*, were the most abundant representing 28.49%, 10.28% and 6.12% of the ARGs detected, respectively. Other highly abundant ARGs were *mel* (9.09%), *cfxA2* (7.17%), *lnuC* (6.11%), and *bla*_OXA-608_ (5.04%) (Additional file 2, Figure S4).

**Fig. 6:**
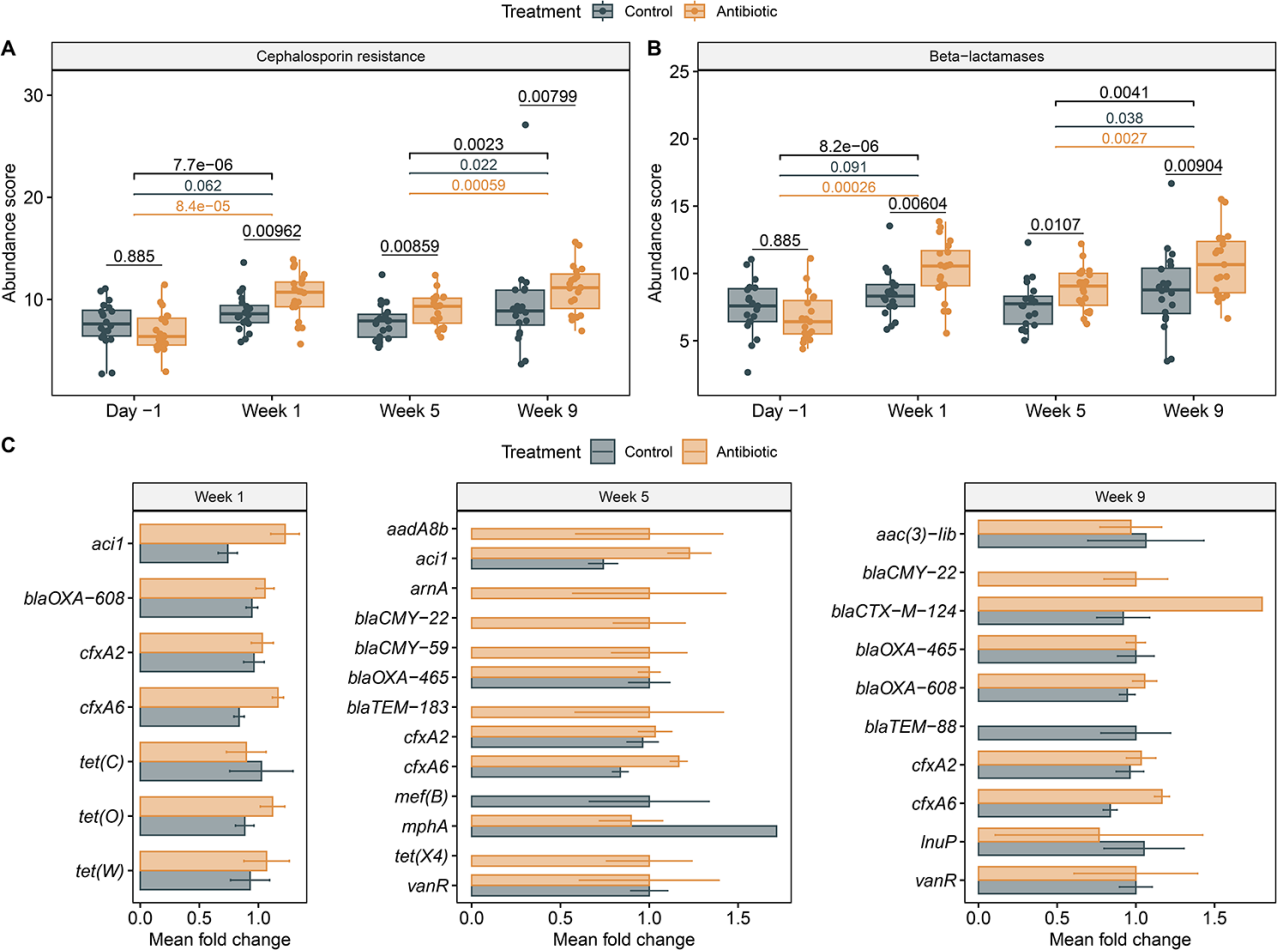
Effects of IMM ceftiofur treatment on the fecal resistome of cattle. Boxplots show the abundance score for genes encoding **A**) resistance to cephalosporins; and **B**) β-lactamases conferring resistance to cephalosporins. **C**) Differentially abundant ARGs are shown after ceftiofur treatment with the mean fold-change and standard error per group. The median, lower, and upper quartiles are shown in each boxplot with the whiskers representing extreme values in the distribution. *P*-values were calculated using the paired Wilcoxon signed rank test to compare treatment groups within a sampling point and between sampling points within the same treatment group; significant *p*-values are shown for all cows (black), control cows (grey), and cows treated with ceftiofur (orange)

Importantly, a persistent increase in the abundance of genes encoding cephalosporin resistance was identified in the ceftiofur-treated cows relative to the controls (Fig. 6A). The treated cows, for example, had a significantly greater abundance score at weeks 1, 5, and 9 relative to the control cows (Wilcoxon signed rank test, *P* < 0.008 at each week) In comparison to the baseline measurement taken on day − 1, the treated cows also had an increased abundance of cephalosporin resistance genes one week after IMM treatment (Wilcoxon signed rank test, *P* < 0.01). A significant increase, however, was not observed in the control cows (Wilcoxon signed rank test, control, *P* = 0.062). In all, the greatest abundance of cephalosporin resistance genes was found in week 9, which was significantly higher than in week 5 for both groups (Wilcoxon signed rank test, control, *P* = 0.022; antibiotic-treated, *P* = 0.0006). Genes important for cephalosporin resistance encoded antibiotic efflux pumps or were important for inactivation and reduced permeability to the drug. Antibiotic inactivation by β-lactamases was the main mechanism of resistance observed for the cephalosporins, showing a persistent increase in β-lactamase (*bla*) genes in the treated versus control cows (Fig. 6B). The *bla* genes encoding ESBL production, *aci1*, *cfxA2*, and *cfxA6*, were among those that increased over the sampling period (Additional file 1, Table S7). Although controls also had these ESBL genes, more were identified only in the antibiotic-treated group at week 5, including *bla*_CMY-22_ and *bla*_CMY-59_ (Fig. 6C). Additionally, co-selection of other ARGs such as *aadA8b*, *tet(X4)*, and *arnA*, was observed in weeks 5 solely in the ceftiofur-treated cows. These ARGs confer resistance to aminoglycosides, tetracyclines, and peptides, respectively.

### The plasmidome, virulome, and virome varied between groups

**Fig. 7:**
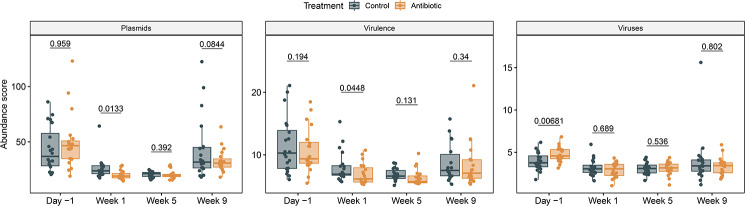
Effects of intramammary (IMM) ceftiofur treatment on the abundance of plasmids, virulence factors and viruses. The median, lower, and upper quartiles are shown in each boxplot with the whiskers representing extreme values in the distribution. *P*-values were calculated with the Wilcoxon signed rank test to compare the ceftiofur-treated and control cows within a sampling point

The normalized abundance of plasmids and virulence genes was significantly lower in ceftiofur-treated cows in the first week after treatment compared to the controls (Wilcoxon signed rank test, *P* < 0.05; Fig. 7); however, the number of observed features was similar between groups (Wilcoxon signed rank test, *P* > 0.05). Despite the higher abundance of viruses identified prior to treatment in the antibiotic-treated group, no differences were observed at later time points. Nonetheless, there were significant differences in the mean plasmidome and virulome composition over time, with days −1 and week 9 as well as week 1 and week 5 forming two clusters in the PCoA (PERMANOVA, plasmids, *F* = 28.60, *R*^*2*^ = 0.36; virulence genes, *F* = 4.79, *R*^*2*^ = 0.085; all, *P* = 0.001) (Additional file 2, Figures S5A and S5B). The virome composition also showed significant differences over time, but clear clusters were not observed in the PCoA (PERMANOVA, *F* = 9.2, *R*^*2*^ = 0.15, *P* = 0.001) (**Figure S5C**). These analyses suggest that IMM ceftiofur lowered the abundance of plasmids and virulence factors in the short term.

### Multiple bacterial hosts had phenotypic or genotypic resistance to β-lactams

*Culture-based identification.* Among 882 Gram-negative bacterial isolates resistant to ceftiofur, 146 were preserved for further analyses; 72 were recovered from control cows and 74 from ceftiofur-treated cows. A maximum of 4 CFUs were selected per sample based on differences in morphology and lactose fermentation on MAC media. Biochemical assays classified 94 isolates as *E. coli*, 25 as other members of Enterobacteriaceae, and 27 as non-Enterobacteriaceae.

*β-lactamase-carrying contig (BCC) characterization.* Among all 40 cows, 158 *bla* alleles conferring resistance to cephalosporins were identified in the fecal resistome. For those contigs with a length ≥ 500 bp, 287 contigs carried β-lactamase genes, which had an average size of 3,446.54 bp (± 3,544.4 bp). These BCCs carried *aci1* (*n* = 13), *cfxA2* (*n* = 219), *cfxA3* (*n* = 5), *cfxA4* (*n* = 32), *cfxA5* (*n* = 2), and *cfxA6* (*n* = 16). The average coverage estimate was 7.53 (± 4.2), while the number of 77 bp k-mers was 30892.6 (± 35537.02). Co-localization of MGEs was identified in 166 (57.84%) of the contigs. β-lactamase genes *cfxA2*, *cfxA4*, and *cfxA6* were commonly found in those contigs containing MGEs for conjugation. These elements include *mobN*, *mobB*, *traC*, and *HMPREF1204_00020*, which encodes a DNA primase (EC2.7.7.-) that was linked to multidrug resistance in *Bacteroides* in a prior study [50]. Despite these findings, the taxonomic classification of the contigs with CAT was only possible for 20 contigs. These included genes encoding ACI-1 in Gammaproteobacteria, CfxA2, CfxA4, and CfxA6 in Bacteroidetes, EC-5 in *Treponema*, OXA-659 in Campylobacterales, SHV-160 in Proteobacteria and Bacteroidetes, and TEM-116 and TEM-183 in Enterobacteriaceae. For 18 of the 20 contigs, taxonomic assignments using CAT were based on a single ORF. Two contigs were exceptions: one classified as Aeromonadales based on 2 ORFs, and another as *Bacteroides xylanisolvens* based on 14 ORFs.

*RGI host assignations.* The resistomes and variants database (CARD) also provided taxonomic assignations for various ARG alleles (Additional file 1, Tables S8-S9). Among the most abundant β-lactamases, the *cfxA2* sequences were assigned to *Phocaeicola* (45.94% of the allele reads), *Bacteroides* (38.8%), *Prevotella* (1.16%), *Parabacteroides* (9.65%), and *Butyricimonas* (4.44%). The *bla*_CFX-A6_ sequences were mostly assigned to uncultured organisms (97.55%) and *Bacteroides* (2.45%), whereas *aci1* was only assigned to *Acidaminococcus fermentans*. Genes encoding CMY-22 and CMY-59, which were detected only in the antibiotic-treated cows, were assigned to *E. coli* and *Klebsiella pneumoniae*, respectively. Other highly abundant β-lactamase genes were common in cows from both treatment groups including those encoding OXA-608, which was assigned to *Campylobacter jejuni*, and SHV-160 assigned to *Klebsiella pneumoniae*.

*Correlation networks*. Correlations between β-lactamase genes and plasmids, phages, and virulence genes showed their potential ecological associations in the fecal microbiota. *E. coli* was the most common host of plasmids, phages and virulence factors correlated with β-lactamase genes, followed by *Klebsiella*, *Salmonella* and other Enterobacteriaceae (Additional file 2, Figure S6). The genera correlated with β-lactamase genes, however, were primarily from phyla Firmicutes, Bacteroidetes, Actinobacteria, and Proteobacteria (Additional file 2, Figure S7). No significant differences were observed in centrality measures between treatment groups at any time point, suggesting that the co-occurrence of β-lactamase genes with other genes and taxa was ecologically similar in both groups.

## Discussion

It was estimated that ~ 90% of dairy farms use IMM β-lactam antibiotics during the dry-off period to treat mastitis [5–7] despite the possibility of selecting for resistant bacterial populations. Of great concern is the emergence and selection of ESBL-producing Enterobacteriaceae, which are considered a serious public health threat [1, 2]. Although the effect of IMM ceftiofur treatment has been studied in the milk microbiota, including five days with IMM 125 mg/day [51, 52] and a single application of 2 g of CHCL [53], the impact of this treatment on the gut microbiome had not been elucidated. Through this study, we have demonstrated persistent effects on the fecal microbiome due to a single 2 g dose of IMM ceftiofur via culture-based analyses and metagenomics. Compared to the controls, the antibiotic-treated cows had altered microbial profiles and a greater abundance of β-lactam resistance genes that increased in abundance over the dry-off period; a subset also had elevated concentrations of cultivable ceftiofur-resistant Gram-negative bacteria.

Following subcutaneous treatment, a prior study showed that Holstein steers had higher concentrations of CHCL in the gastrointestinal tract compared to ceftiofur crystalline-free acid (CCFA) [9], though only CCFA resulted in decreased fecal *E. coli* concentrations for up to two weeks. Similarly, parenteral ceftiofur treatment resulted in lower fecal *E. coli* concentrations for 3 days [16] and up to a month post-treatment [17]. In the latter study, systemic ceftiofur administration resulted in a significant increase in the level of ceftiofur-resistant Enterobacteriaceae, though the concentrations returned to baseline levels after one week [17]. Consistent with these findings, we observed a reduction in the total number of Gram-negative bacteria in the treated cows one week after IMM ceftiofur treatment when compared to controls as well as enhanced recovery of Gram-negative bacteria with resistance to ceftiofur for two weeks post-treatment. Re-emergence of ceftiofur resistance in the Gram-negative bacteria was also observed at 9 weeks (pre-calving) in both the treated and control animals, which is similar to results from another study [17]. Herein, this increase was associated with sampling period and ambient temperature as well as lactation phase, which involves specific dietary requirements and physiological states. Ambient temperature affects the cows’ metabolism and immune response [54], which can impact the gut microbiota, including the population of Gram-negative bacteria. In fact, warmer temperatures have been linked to higher levels of fecal shedding of Shiga-toxin producing *E. coli* in dairy cattle [55]. Diet, which is linked to lactation phase, can also impact the gut microbiota. Indeed, a diet containing higher levels of metabolizable energy can promote the growth of certain bacteria, possibly including certain Gram-negative species, when given to lactating and fresh cows [56]. Dietary changes can also influence the pH level in the gut, which can further impact bacterial growth and antibiotic resistance [57, 58]. Other factors that could contribute to the expansion of resistant Enterobacteriaceae populations include environmental acquisition of resistant strains, increased frequency of horizontal gene transfer, peri-parturient immune suppression, or increased contact with personnel. Regardless, it is important to note that in vitro bacterial quantifications do not distinguish between acquired and intrinsic antimicrobial resistance. Future studies should therefore focus on isolating the resistant strains for characterization using biochemical tests and whole-genome sequencing, which can define the genetic mechanisms of resistance as well.

Following IMM ceftiofur treatment, a lower abundance score and diversity of taxa was detected in the fecal microbiota, which was also observed for plasmids and virulence genes. Conversely, a higher abundance of ARGs was observed in the antibiotic-treated cows one week following IMM treatment when compared to the control cows. Because this difference was not observed in the subsequent time points, it suggests the temporary selection of resistant bacterial populations. Intriguingly, *Campylobacter* and *Bifidobacterium* were more abundant in the ceftiofur-treated cows as compared to controls, which is not surprising given that most *Campylobacter*, with the exception of *C. fetus*, have intrinsic resistance to third-generation cephalosporins [59]. In fact, nine β-lactamase genes were associated with *Campylobacter* including *bla*_*OXA-608*_, which was one of the most abundant ARGs detected.

Despite the temporary increase in ARG normalized abundance and diversity observed one week after ceftiofur treatment, a subset of critically important genes persisted. Importantly, the antibiotic-treated cows had an exclusive and persistent increase in the abundance of ESBL genes (e.g., *aci1*, *cfxA*, and *bla*_CMY_) in the fecal resistome at each of the subsequent time points examined. Although increases in the abundance of ESBL genes following parenteral ceftiofur treatment have been reported, no prior studies have examined the effect of IMM treatment. Steers receiving subcutaneous CCFA, for example, had a higher abundance of bacterial isolates harboring *bla*_CMY-2_ up to 4 days post-treatment, which resulted in co-selection of isolates containing *tet(A)* and *bla*_CMY-2_ after a subsequent chlortetracycline treatment for up to 26 days [18]. Similarly, Holstein cows treated with systemic CCFA had a higher abundance of genes encoding CfxA β-lactamases three days after treatment [60], while other studies reported an increase in *bla*_CMY-2_ in cattle feces for up to 10 days post-treatment when pure cultures were analyzed [16, 61]. Our findings are consistent with these previous studies and underscore the need for the judicious use of third-generation cephalosporins in livestock. Furthermore, they highlight how continuous monitoring is needed to understand how ARGs are maintained in dairy cattle and the farm environment.

Although the abundance of ESBL genes was higher in the ceftiofur-treated cows across the sampling period, an increase in cephalosporin-resistant bacterial populations (CFUs) was not observed. This discrepancy between the culture-based and sequencing methods could be attributed to the oxygenic environment and/or media used for cultivation. The hindgut microbiota is composed predominantly of anaerobic bacteria; thus, aerobic and microaerophilic conditions used for the quantification of Gram-negative and Gram-positive bacteria could only capture a fraction of the microbiota. Bacteroidetes members like *Prevotella* and *Bacteroides*, for example, are common Gram-negative anaerobes residing in the hindgut. Because these members were commonly found to carry genes encoding CfxA ESBLs [62], the resistant CFUs observed likely underestimate the actual levels of resistance, particularly given the high abundance of *cfxA* alleles detected. Likewise, *aci1* was the second most abundant ESBL gene and was previously reported in the Gram-negative Firmicutes *Acidaminococcus* [63] and Gram-positive genus *Bifidobacterium* [64]. In fact, a prior study identified that *Bacillus*, *Bacteroides*, *Eubacterium*, *Bifidobacterium*, *Clostridium* and *E. coli* are important degraders of ceftiofur in the bovine gut [65]. These findings suggest that the increased abundance of ESBLs following IMM ceftiofur treatment were linked to changes in the abundance of anaerobic bacteria, which is consistent with our host tracking analyses.

Indeed, identifying bacterial hosts and MGEs associated with β-lactam resistance genes in cattle feces is critical for developing new interventions, understanding the ecology of potential resistant threats that may emerge in farm environments, and defining risks associated with carriage of specific genes. As described herein, one approach to classify bacterial hosts is by identifying contigs or metagenome-assembled genomes containing genes encoding known β-lactamases. While culture identification of the resistant bacteria indicated 64% of the isolates were *E. coli*, metagenomic analyses showed that β-lactamase genes were mainly associated with commensal bacteria. A significant association was also identified between *bla*_CfxA_ and plasmid sequences, suggesting that horizontal gene transfer plays a key role in the acquisition of CfxA β-lactamase genes, particularly for members of phylum Bacteroidetes. Evidence of the relationship between Enterobacteriaceae and genes encoding the CMY, CTX, OXA, and TEM β-lactamase families was supported through the RGI analysis and co-occurrence networks showing correlations between these genes and plasmid sequences. Together, these results demonstrate the importance of horizontal gene transfer in the dissemination of antibiotic resistance within bacterial communities, particularly among members of the Bacteroidetes phylum and within the Enterobacteriaceae family.

Intriguingly, the abundance of Actinobacteria was significantly higher on day −1 compared to the subsequent time points. The most abundant family belonging to phylum Actinobacteria was Bifidobacteriaceae, which was represented mainly by the genus *Bifidobacterium*. Bifidobacteriaceae are implicated in the utilization of oligosaccharides in the colon resulting in the production of volatile fatty acids (VFAs) [66]. Differences in the composition of the fecal microbiota, primarily caused by the abundance of Actinobacteria observed on day −1 could be associated with differences in the diet. During late lactation, higher levels of dry matter intake and metabolizable energy as well as protein are consumed by cows compared to the dry off (weeks 1–7) and fresh (week 9) periods. However, further analyses of microbial metabolic pathways and metabolite composition are necessary to better explain how differentially abundant taxa may impact cow performance.

Although this study is the first to describe the impact of IMM ceftiofur treatment on the gut microbiota, it is important to highlight a few limitations. For instance, current resistome databases do not include all known ARGs from cattle samples and hence, novel resistance determinants may remain unclassified. Moreover, the identification of species and ARGs can be limited by a low number of metagenomic reads, as sequencing depth of ≥ 50 million reads is needed for complex microbial communities such as those residing in the bovine gut [67]. Since the proportion of microbial phyla and ARG classes was shown to be constant across various sequencing depths [67], we were able to detect the predominant and differential metagenomic features in this analysis. The shallow sequencing depth and short DNA segments (150 bp) examined, however, may have reduced our ability to accurately classify the bacterial hosts within each BCC since flanking regions are often not included. Such issues could have also contributed to the discordance observed between the sequence- and culture-based methodologies. Consequently, future work involving use of third-generation sequencing platforms that sequence ultralong DNA segments such as the PacBio (40–70 Kbp) or Oxford Nanopore Technologies (> 100 Kbp), is needed for confirmation and characterization of these regions [68]. Since the identification of differentially abundant features, including bacterial taxa and genes, tends to vary across bioinformatic pipelines, we applied three different approaches but only reported those features with significant *p*-values using at least two pipelines, as suggested previously [69]. Consequently, our analyses highlight those microbiome features, genes and taxa, that are most impacted by IMM ceftiofur treatment.

## Conclusions

One application of IMM ceftiofur (2 g) at dry off contributed to alterations in the fecal gut resistome, which resulted in an increase in the abundance of genes encoding resistance to cephalosporins and ESBLs in the treated versus control cows. Importantly, these genes were maintained in the cow gut at high levels for the 9-week sampling period. Clinically important ESBL genes were mainly associated with Bacteroidetes and Enterobacteriaceae hosts as well as plasmid sequences, illustrating how ESBL-producing pathogens emerge and are selected for in this niche. While most of the cows given the prophylactic IMM ceftiofur treatment did not have altered microbiota compositions compared to the control cows, 25% had an increased level of ceftiofur-resistant Gram-negative bacteria for up to 2 weeks post-treatment. Indeed, the recovery of resistant CFUs was 14× greater in the antibiotic-treated versus control cows at week 2. These findings demonstrate significant variation in the fecal shedding levels of cultivable bacterial populations across animals in this herd, which could be linked to selective factors such as diet, temperature, and lactation phase. Future studies should therefore focus on understanding the association between shedding and the dissemination and persistence of antibiotic resistance determinants in dairy farm environments across geographic locations.

### Electronic supplementary material

Below is the link to the electronic supplementary material.

Supplementary Material 1

Supplementary Material 2

## Data Availability

The paired-end metagenome raw reads analyzed in this study are available in the NCBI repository under BioProject PRJNA825520 (Samples: SRR25542980-SRR25542689; Blanks: SRR25542875-SRR25542799). Datasets and analyses supporting the conclusions are available in the GitHub repository, [Effects of intramammary ceftiofur in the cow’s metagenome in https://github.com/karla-vasco/metagenome_cows_IMM-ceftiofur].

## Abbreviations

Amp^R^: Ampicillin resistant
Amp^S^: Ampicillin susceptible
ANCOM-BC: Analysis of compositions of microbiomes with bias correction
ARG: Antibiotic resistance gene
ATCC: American Type Culture Collection
BCC: β-lactamase gene carrying contig
BLA: β-lactamase gene
BLASTP: Protein Basic Local Alignment Search Tool
CARD: Comprehensive Antibiotic Research Database
CAT: Contig Annotation Tool
CCFA: Ceftiofur crystalline-free acid
CefR: Ceftiofur resistant
CefS: Ceftiofur susceptible
CFB: Cytophaga, Fusobacterium, and Bacteroides
CFU: Colony forming unit
CHCL: Ceftiofur hydrochloride
CLSI: Clinical and Laboratory Standards Institute
CNA: Columbia Nalidixic Acid Agar
DHIA: Dairy Herd Improvement Association test
DNA: Deoxyribonucleic acid
ESBL: Extended spectrum β-lactamase
IMM: Intramammary
LDA: Linear discriminant analysis
LEfSe: Linear Discriminant Analysis Effect Size algorithm
PCoA: Principal coordinate analysis
PERMANOVA: Permutational multivariate analysis of variance
RGI: Resistance Gene Identifier
SCC: Somatic cell count
VFDB: Virulence Factor Database
