# Supplementary material for: Persistent effects of intramammary ceftiofur treatment on the gut microbiome and antibiotic resistance in dairy cattle
Source: Anim Microbiome. 2023 Nov 9;5:56. doi: 10.1186/s42523-023-00274-4 (PMC10636827; doi:10.1186/s42523-023-00274-4)
Supplement: Supplementary file 2 — Supplementary Material 2 [file 42523_2023_274_MOESM2_ESM.pdf]

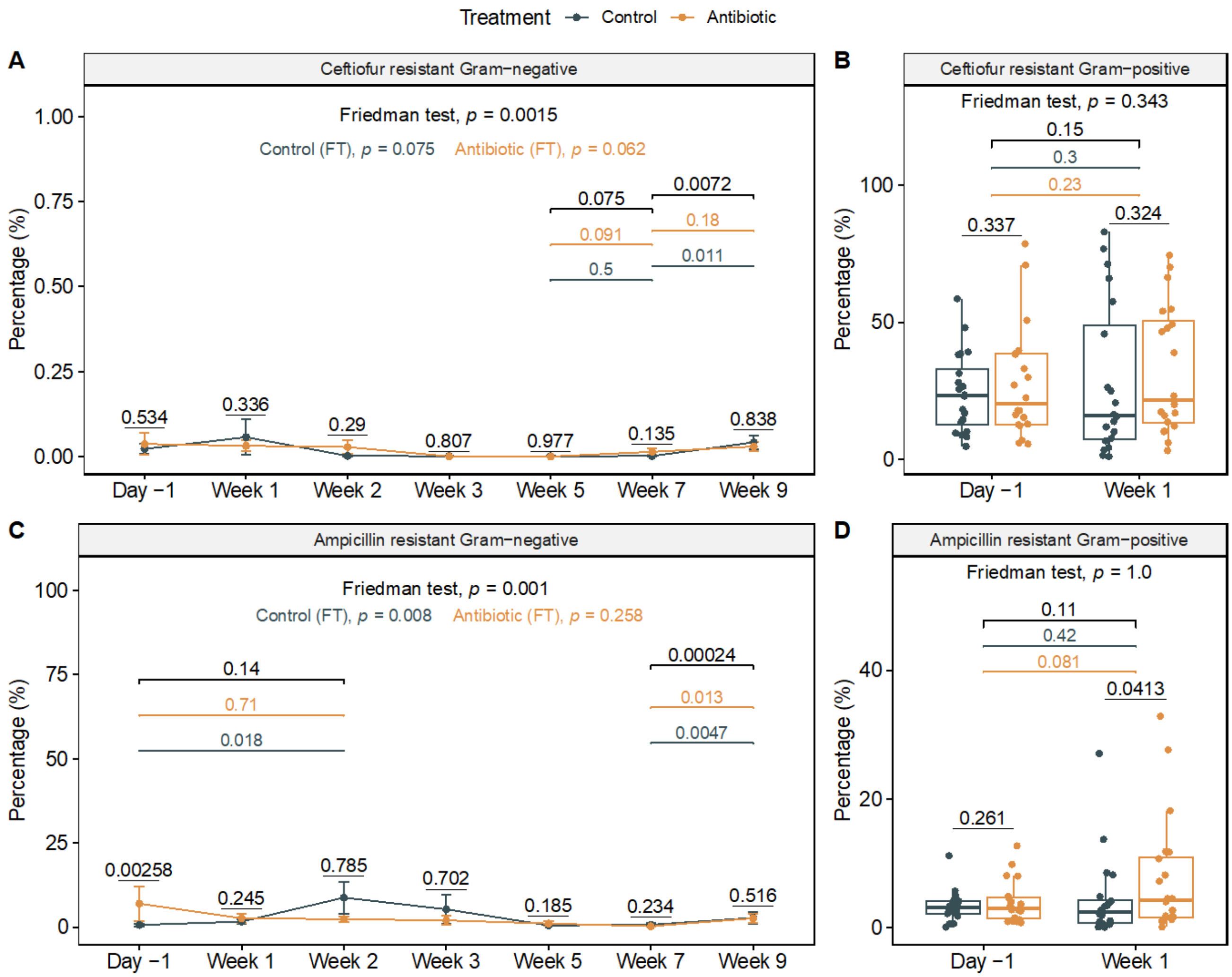

Figure S1. The proportion of resistant bacteria recovered. The percentages of **A**) Gram-negative and **B**) Gram-positive bacteria with resistance to ceftiofur, and **C**) Gram-negative and **D**) Gram-positive bacteria with resistance to ampicillin were calculated based on the total number of CFU/g of feces per sample. Numbers are plotted before (Day -1) and after treatment for Gram negative bacteria through 9 weeks and for Gram-positive bacteria after 1 week. Line plots show the means and standard error bars with sample counts represented as dots for the ceftiofur-treated (orange) and control (black) cows. P-values were calculated with paired Wilcoxon test to compare treatment groups within a sampling point and the same group across two samplings. Boxplots indicate the median, lower and upper quartiles, and the whiskers represent extreme values in the distribution. The per animal variability over time was calculated with Friedman's rank-sum test (FT), which is shown per treatment group for Gram-negatives. Significant p-values between sampling points are shown for all animals (black) as well as the control (grey) and antibiotic-treated (orange) animals.

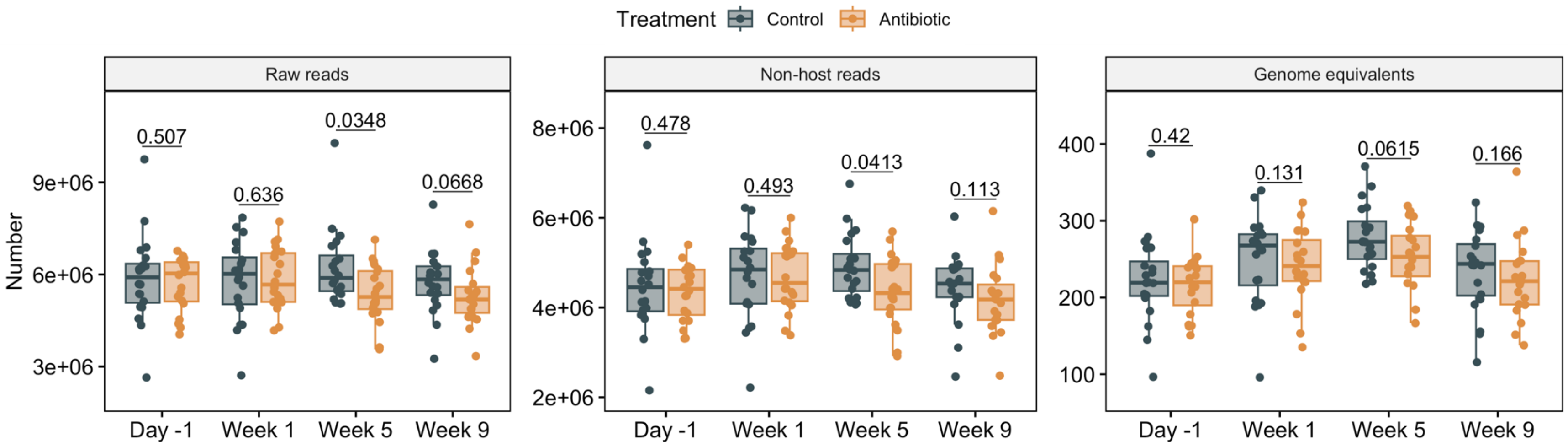

Figure S2. Metagenomic sequencing metrics: raw reads, non-host reads and genome equivalents. Boxplots indicate the median, lower and upper quartiles, and the whiskers represent extreme values in the distribution. P-values were calculated with paired Wilcoxon test to compare treatment groups within a sampling point. Genome equivalents were calculated with MicrobeCensus.

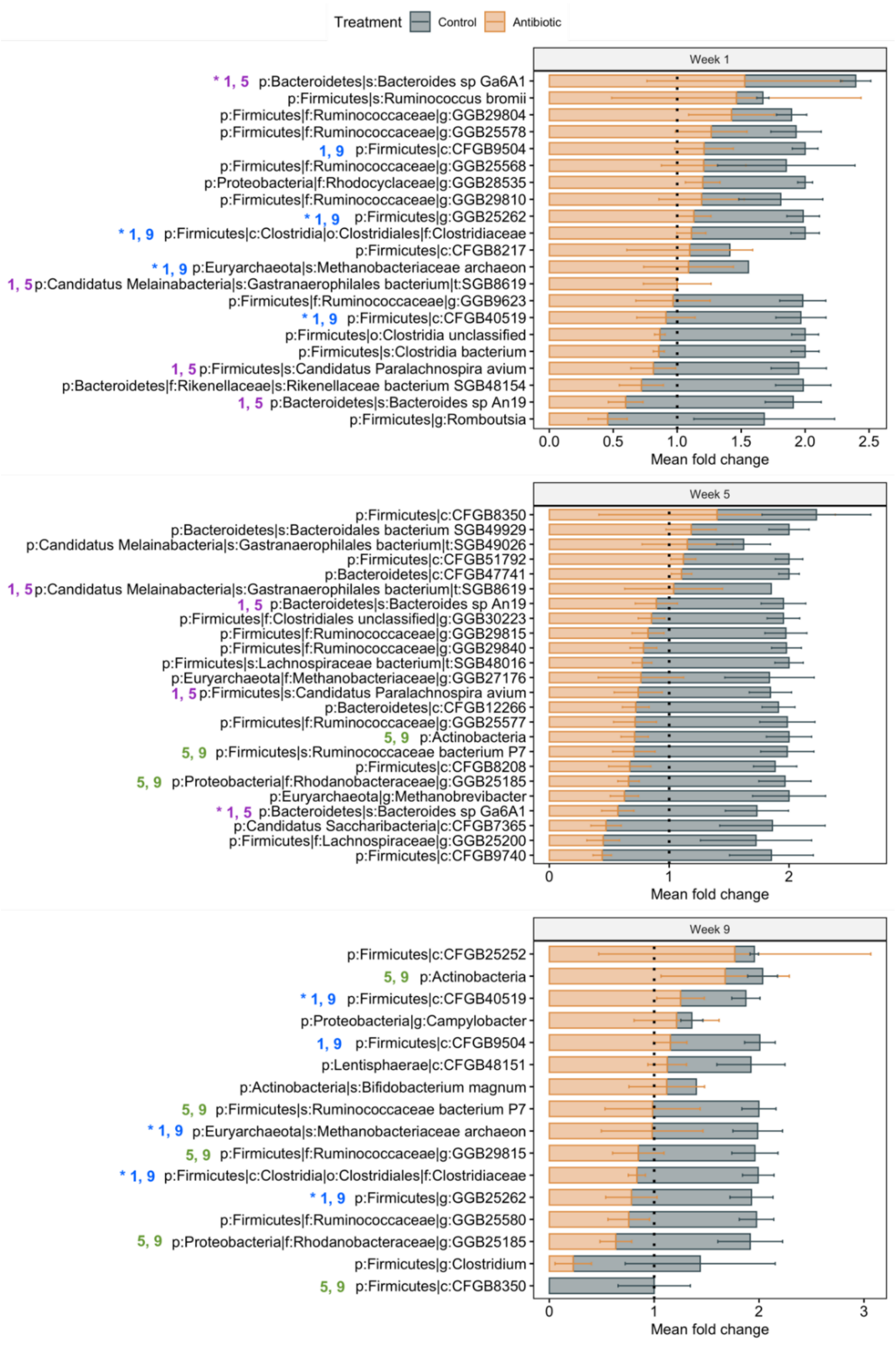

Figure S3. Differentially abundant microbial taxa identified after IMM therapy showing the mean fold change and standard error per treatment group. Taxa that was significantly different in more than one point is indicated in blue for those observed in weeks 1 and 9, purple in weeks 1 and 6, and green in weeks 5 and 9. An asterisk indicates that a specific taxon was identified in two different time points but with opposite overrepresentation in a determined treatment group.

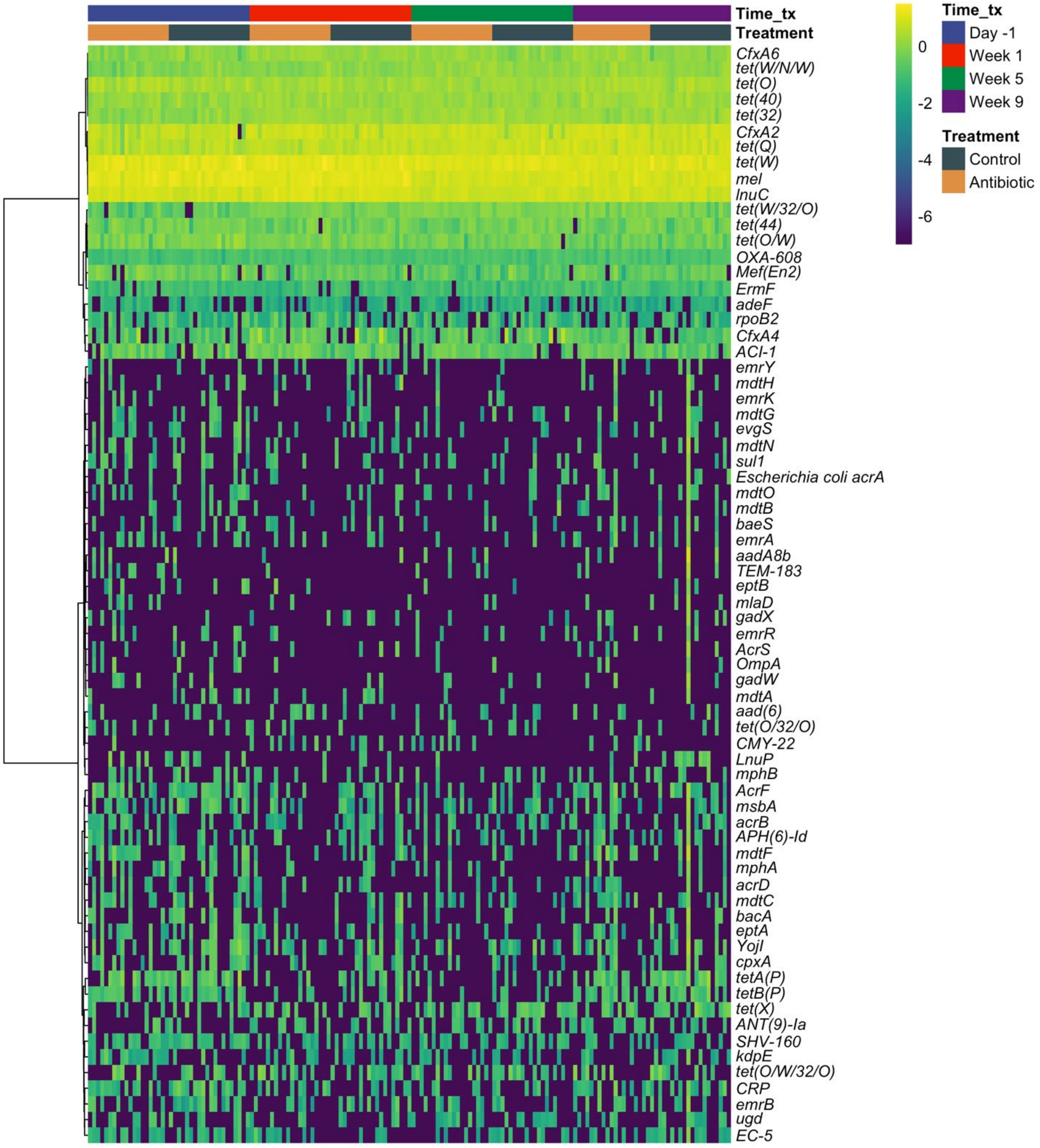

Figure S4. Heat map of the 70 most abundant ARGs present in the fecal metagenome of cows. Samples are organized by time point and treatment group. ARGs are clustered using the hierarchical clustering method Ward D2. The values represent logarithm 10 of the normalized abundance.

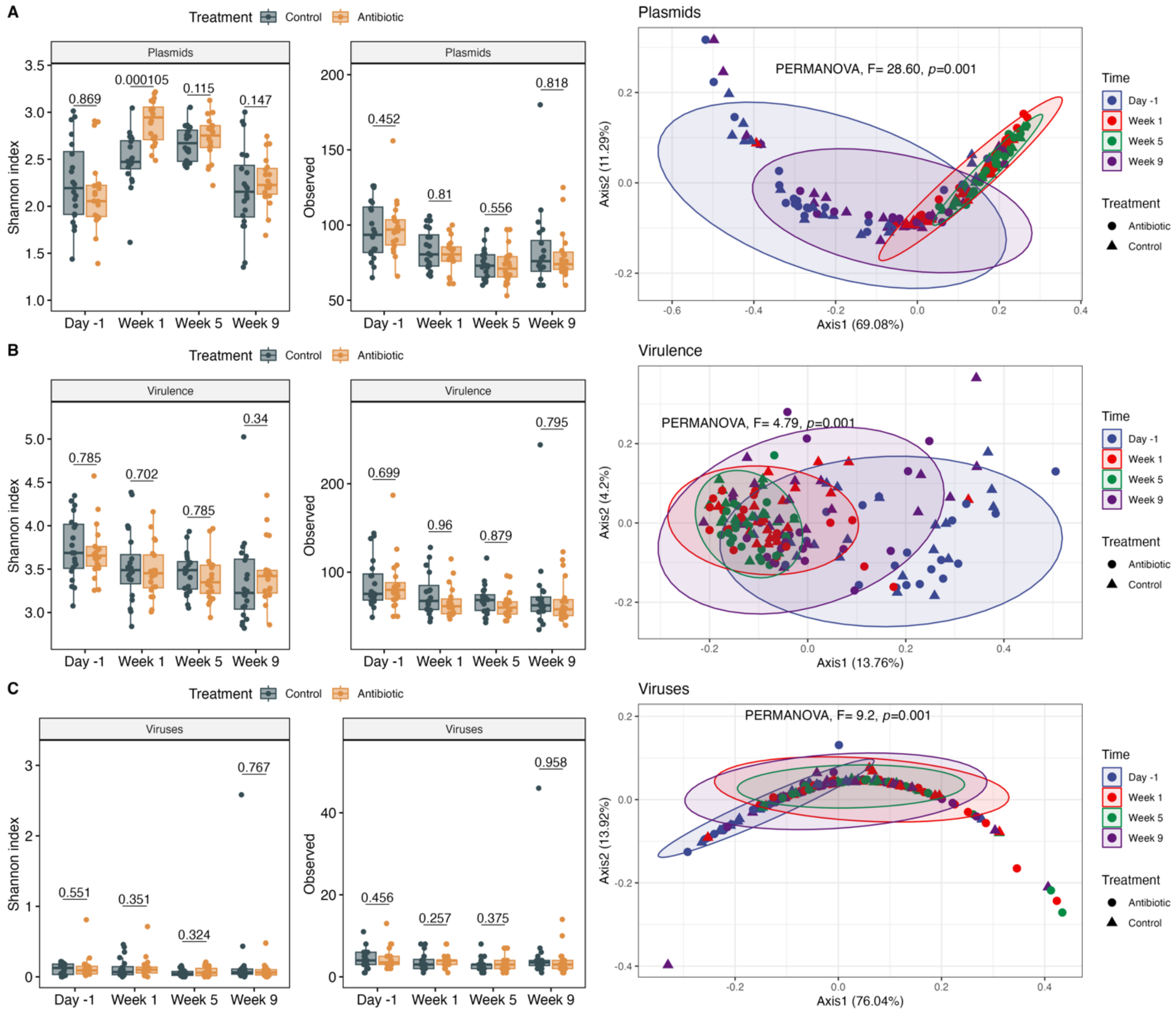

Figure S5. Plasmid (A), virulence genes (B) and viruses (C) alpha and beta diversity in the fecal metagenome of dairy cows. The Shannon index and observed features are indicated as boxplots that include the median, lower and upper quartiles, and the whiskers represent extreme values in the distribution. P-values were calculated with paired Wilcoxon test to compare treatment groups within a sampling point. PCoA of the Bray-Curtis dissimilarity is shown and clustered by time-point and treatment. Ellipses in the PCoA include 90% of the samples and PERMANOVA was calculated for differences between time points.

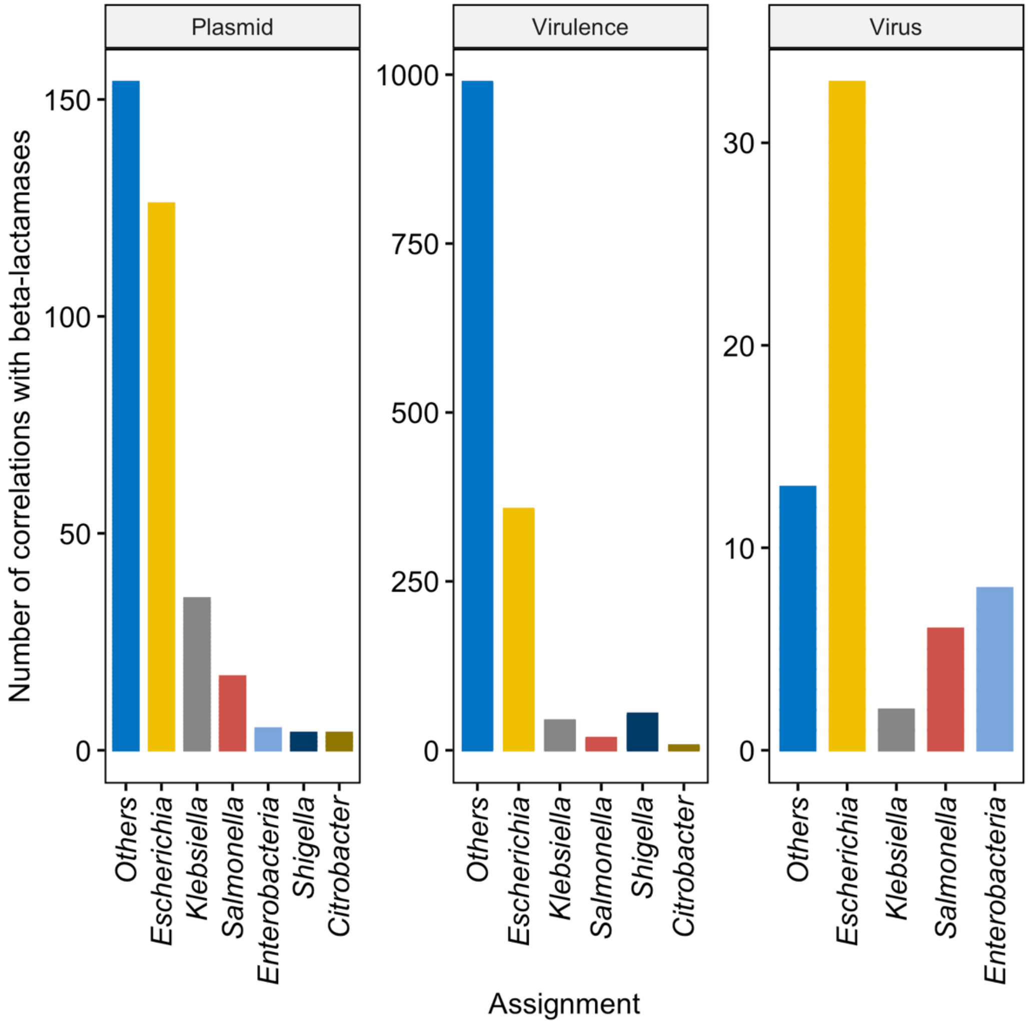

Figure S6. Taxonomic assignment of plasmids, virulence factors and viruses correlated with β-lactamases. The number of correlations identified between β-lactamases conferring resistance to cephalosporins are represented in the Y axis. Assignations were summarized at the genus level. The network included correlations ≥ 0.75 ($P < 0.01$) calculated in a matrix with the normalized abundances of all samples ($n = 159$).

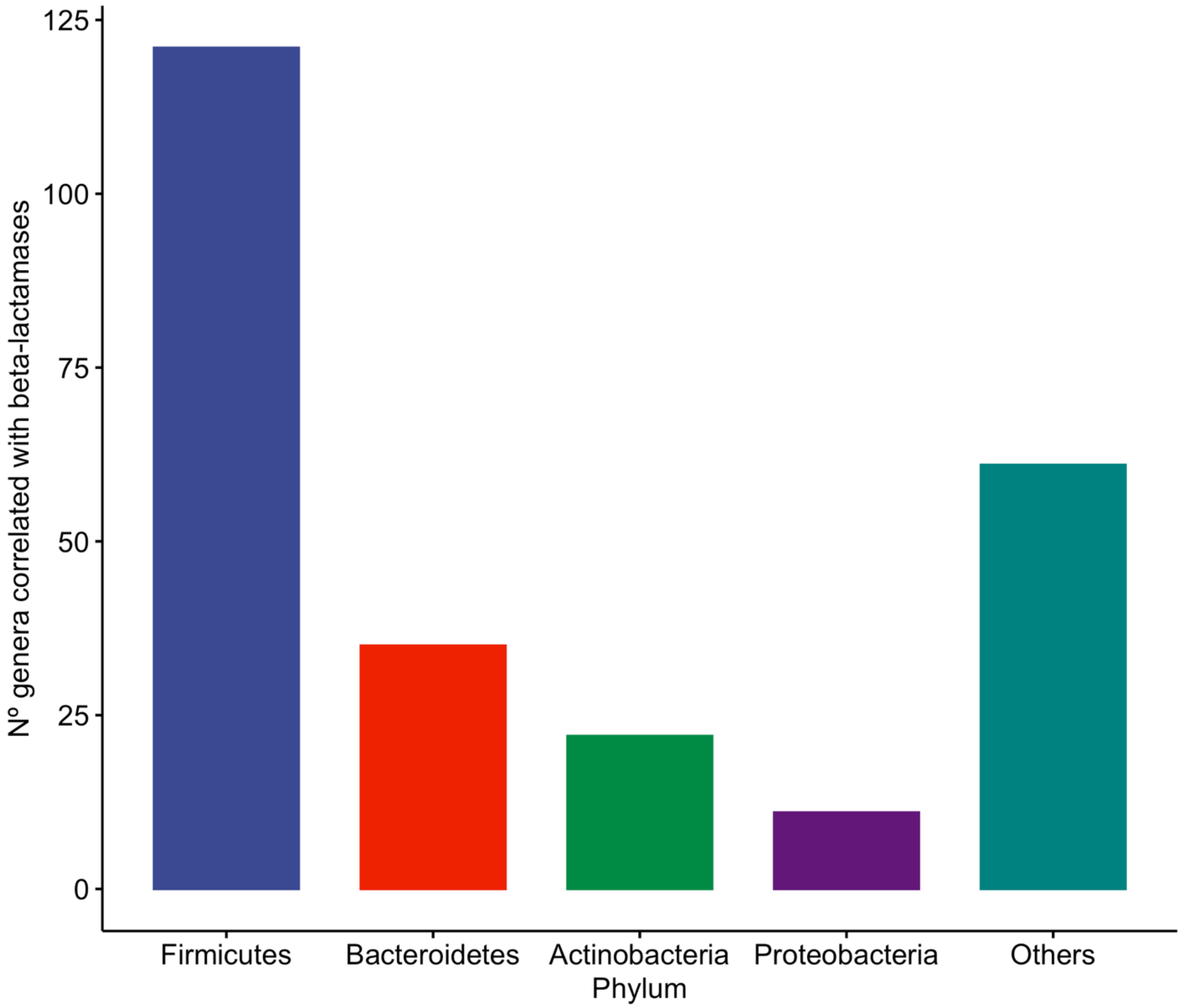

Figure S7. Taxonomic assignment of genera clustered at the phylum level that was correlated with β-lactamases. The number of correlations identified between β-lactamases conferring resistance to cephalosporins are represented in the Y axis. The network included correlations ≥ 0.75 (*P* <0.01) calculated in a matrix with the normalized abundances of all samples (*n* = 159).
